# Warming temperatures increase close encounters between two top predator species via changes in spatial behaviour

**DOI:** 10.1186/s40462-026-00635-z

**Published:** 2026-04-11

**Authors:** Kasim Rafiq, Anna C. Nisi, Neil R. Jordan, Krystyna A. Golabek, J. W. McNutt, Alan Wilson, Laura Prugh, Dikatholo Kedikilwe, Briana Abrahms

**Affiliations:** 1 Center for Ecosystem Sentinels, Department of Biology, University of Washington, Seattle, USA; 2 Botswana Predator Conservation, Wild Entrust, Maun, Botswana; 3 Centre for Ecosystem Science, School of BEES, University of New South Wales, Sydney, Australia; 4 Taronga Conservation Society Australia, Sydney, Australia; 5 Structure & Motion Lab, Royal Veterinary College, London, UK; 6 School of Environmental and Forest Sciences, University of Washington, Seattle, USA; 7 Sankoyo Tshwaragano Management Trust, Sankoyo, Botswana; 8 Round River Conservation Studies, Utah, USA

**Keywords:** Bio-logging, Cheetahs, Lions, African wild dogs, GPS collars, Temperature, Interspecific encounters, Large predators, Competition, Space use, Habitat selection

## Abstract

Effectively responding to changing environmental and ecological conditions, such as changing climatic conditions, is crucial for individual survival, population dynamics, and ecosystem structuring. Yet it is often unclear to what extent climate-mediated behavioural adjustments impact potential interspecific dynamics within upper trophic levels, particularly within top predator guilds, such as large mammalian carnivores, and to what extent such environmentally mediated plasticity shapes encounters among top predators. We used the African large predator guild, one of the last remaining functionally intact large predator assemblages, to investigate the impacts of ambient temperature on spatial behaviour and close encounters amongst top predators. Using high-resolution GPS data collected over eight years (2011–2018) from 43 individuals across lions (*Panthera leo*), cheetahs (*Acinonyx jubatus)*, and African wild dogs (*Lycaon pictus)*, we found one of the first empirical examples of changes in close encounters, herein encounters, between top predator species due to temperature variability. The probability of encounters increased under warming temperatures between cheetahs and lions, with encounters 6.6 times more likely following hot (90th percentile) days, compared to median temperature days. Moreover, we found that these responses paralleled changes in habitat selection and spatial overlap between the species rather than with reductions in temporal partitioning. Interestingly, despite functional similarities between cheetahs and African wild dogs, we found no evidence that African wild dog and lion encounter probabilities, nor spatial overlap, changed as a function of temperature, suggesting that species-specific differences in competitor risk sensitivity may buffer African wild dogs from temperature-driven shifts in competition. Our results provide empirical evidence linking climate variability to changes in close encounters among top predators and highlight how temperature-driven behavioural shifts may alter spatial and temporal relationships between species.

**Supplementary Information:**

The online version contains supplementary material available at 10.1186/s40462-026-00635-z.

## Introduction

Effectively responding to changing environmental and ecological conditions, such as changing climatic conditions, is crucial for individual survival, population dynamics, and ecosystem structuring [1–3]. Across taxa, animals must adjust their behaviour in response to multiple, interacting demands, including thermal constraints, foraging requirements, and exposure to risk [4–8]. For example, animals can adjust the timing of their activities to mitigate exposure to periods of high temperatures [9–11] and alter patterns of habitat selection to track key resources and manage their exposure to predation risk [12, 13]. Yet it is often unclear to what extent climate-mediated behavioural adjustments impact potential interspecific dynamics within upper trophic levels, particularly within top predator guilds such as large mammalian carnivores, and to what extent such environmentally mediated plasticity shapes encounters among top predators. For example, shifting environmental conditions may alter the relative importance of different physiological or ecological demands, such as competition risk avoidance, prey capture, or thermoregulation [8, 12, 14], with potentially important consequences for intraguild interactions. Moreover, shifts in species interactions are one of the most common pathways through which climate can threaten population persistence [15–17], underscoring the need to examine how climate variability is likely to alter such dynamics.

Evaluating climate-mediated impacts on animal community dynamics is particularly challenging as it requires data on animal behaviours and their environments for multiple co-occurring species over long timespans, a logistically taxing and expensive endeavour. Across taxa and body sizes, climate variability can impose physiological and ecological constraints that may restructure species distributions, alter interaction strengths, and propagate through food webs, sometimes influencing trophic cascades and shaping community dynamics [18]. These effects can arise both directly, through thermal constraints on animals, and indirectly, by modifying prey capture, resource availability, and other interaction landscapes [19, 20]. Evaluating these dynamics is particularly challenging in studies of free-ranging apex predators, such as large terrestrial carnivores, as their low population densities, large home ranges, and often elusive behaviour increase the difficulties in finding and studying individuals. Indeed, it has been shown across systems that climate variability can impact the movements, fitness, and demography of individual large carnivore species [1, 21, 22]. However, there have been few empirical studies documenting how the spatio-temporal responses of multiple sympatric species to climate variability may come together to mediate community dynamics, such as spatial overlaps between species and encounter rates in large terrestrial carnivores [13]. Encounters between large carnivores can result in negative outcomes, including kleptoparasitism, injury, or mortality, and are therefore actively avoided by some species [12, 23, 24]. Further, in some cases, risk responses to dominant species are not necessarily predicated on actual negative outcomes [23]. Therefore, it is common for studies to use spatial overlaps, attraction or avoidance, and encounters as proxies for competitive dynamics and risk within top species guilds (for example [25–28]), even in the absence of directly measured antagonistic interactions. Such species often play keystone roles within ecosystems, making understanding the impacts of global environmental change on their community dynamics of particular biological and conservation importance [29].

To test the hypothesis that temperature mediates spatial overlaps and close encounters between top predators, we used multi-year GPS tracking data collected across two large predators (cheetah, *Acinonyx jubatus*, and African wild dog, *Lycaon pictus*) and their dominant competitor (lion, *Panthera leo*) in the Okavango Delta, Botswana. This region is characterised by two seasonal periods defined by precipitation patterns, the dry season (median maximum daily temperature: 28.8 °C, range: 16.8–40.7 °C) and the wet season (31.1 °C, range: 19.9–42.1 °C). This is an apt system to study the impacts of climate variables on carnivore community dynamics because it represents one of the last functionally intact guilds of large carnivores [30], each species has been shown to exhibit varying degrees of behavioural plasticity in response to ambient temperature [1, 11, 31, 32], and it has been experiencing rapid environmental change, including rising temperatures [33]. In our system, warmer days are characterised by increases in both daytime and crepuscular temperatures [1], meaning that animals experience elevated thermal conditions across the diel cycle, including during periods of activity. For example, within our system, cheetahs are relatively diurnal and exposed to the warmest parts of the day, while African wild dogs, herein wild dogs, experience elevated temperatures during their crepuscular activity peaks [1, 11]. As such, increases in daily temperature capture relevant variation in thermal conditions during the periods when species are moving, hunting, and making space-use decisions. Furthermore, the African large carnivore guild exhibits intense interspecific competition. Lions are larger and competitively dominant over cheetahs and wild dogs, and their encounters can lead to subordinate competitors losing critical resources such as prey carcasses, suffering injury, or being killed [34–37]. Indeed, lion interactions are a major source of mortality for cheetahs and wild dogs, and in some populations (though not all), are a key factor limiting population densities [34, 35, 38]. As such, cheetahs and wild dogs can often display niche separation across multiple spatio-temporal scales to reduce close encounters with lions [11, 25–27].

We specifically tested how ambient temperatures impacted close encounters between species and examined the spatial and temporal shifts that may mediate such encounters. Fine-scale spatiotemporal encounters with lions can impose behavioural or energetic costs to both cheetahs and wild dogs [34–36]. Our predictions, therefore, focus on how temperature may influence these fine-scale spatial dynamics rather than long-term population dynamics. We predicted that, as heat-sensitive, chasing predators [11, 22, 32], cheetahs and wild dogs would: (i) show strong changes in space use as a function of ambient temperatures, with cheetahs showing the strongest shift in behaviours due to their being a more diurnal species, and would increase their use of shaded habitats [11], (ii) show greater spatial overlap with lions under warmer conditions, for example potentially reflecting reduced opportunities for spatial avoidance when thermally mediated constraints or resource access narrows the range of suitable habitats, and (iii) show increased close encounter probabilities with lions as temperatures increase, for example, because heat or resource constraints concentrate movements at finer spatial or temporal scales, increasing the likelihood of brief co-locations in space and time even without proportional increases in broader scale spatial overlaps. Note, since encounters require animals to be co-located in both space and time, and are not solely determined by broad spatial overlap, our third hypothesis represents an independent expectation of the first two predictions. Temperature can shape animal behaviour across multiple timescales, with some responses occurring immediately and others unfolding over longer periods due to, for example, the time required for animals to move across the landscape. Accordingly, we examined the effects of both same-day and previous-day temperatures to capture rapid and lagged behavioural responses to temperature variability. We expected temperature to have its strongest influence on spatial responses and encounter probabilities on the day following warmer conditions.

## Methods

### Study system

**Fig. 1:**
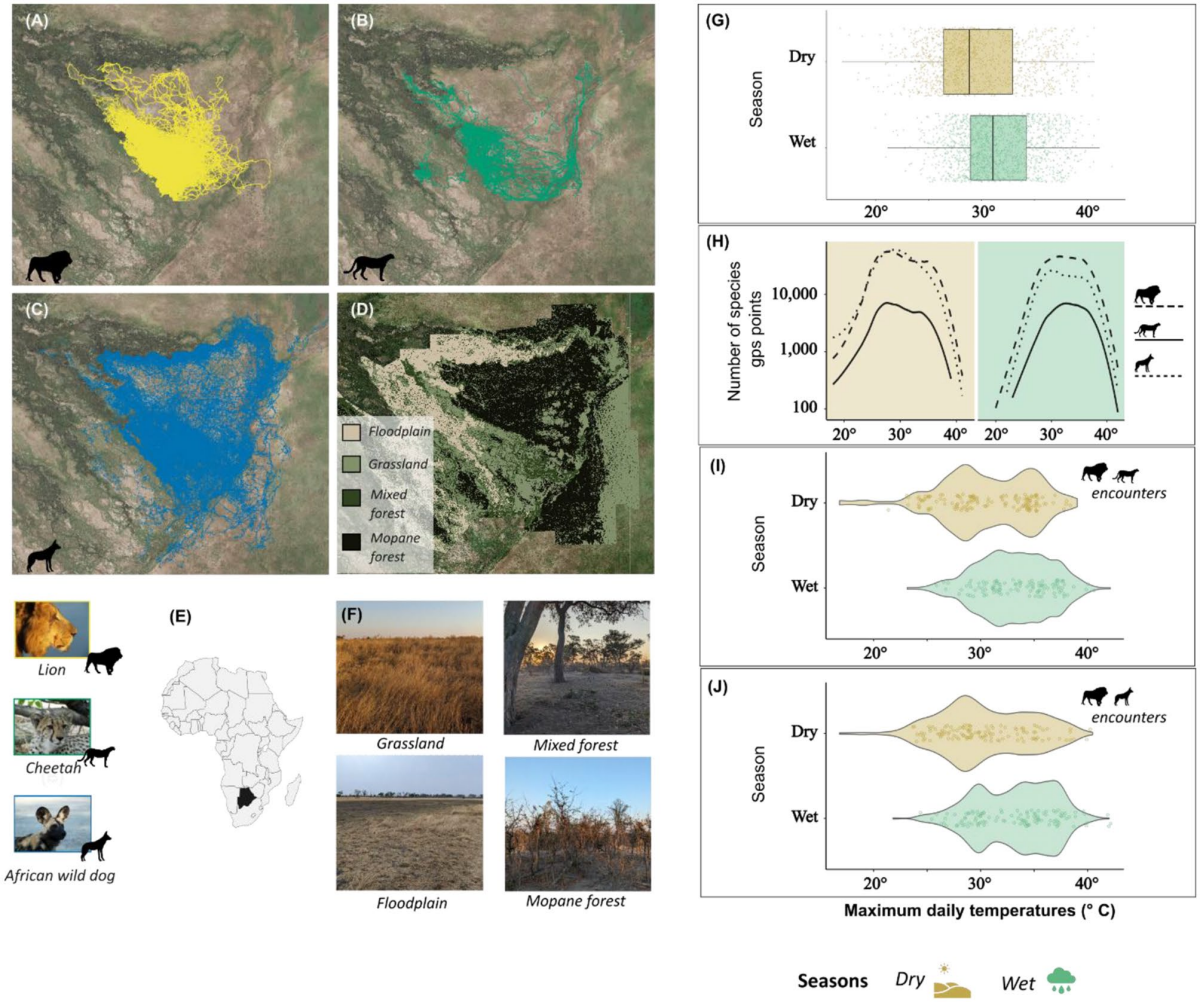
Overview of the study area and data. (**A**-**C**) Spatial extent of the GPS tracking data from lions, cheetahs, and African wild dogs between 2011 and 2018. (**D**) Distribution of key habitat types within the study area. (**E**) Location of the study area in Africa. (**F**) Images showing the differences between key habitat types within the study area. (**G**) Summary of the seasonal maximum daily temperatures (points represent a single maximum daily temperature) across our study period. (**H**) Total number of lion (dashed), cheetah (solid), and African wild dog (dotted) GPS locations collected across seasons and temperature ranges. (**I**-**J**) Distribution of lion-cheetah and lion-African wild dog close encounters, respectively, across maximum daily temperatures (points represent an encounter for a single day)

This study was conducted in the Okavango Delta, Botswana, within a region including Moremi Game Reserve and wildlife management areas primarily used for photographic tourism. The study area encompassed ca. 2,600 km^2^ and comprised four primary habitat types: grasslands, floodplains, mixed woodlands, and mopane forests, defined by the dominant vegetation in the area. Grasslands and floodplains were characterised by a lack of canopy cover, with the dominant vegetation consisting of grass species and with floodplains subject to seasonal flooding [39]. In contrast, mopane forests and mixed woodlands were characterised by the prevalence of *Colophospermum mopane* and various other woody species, respectively, and had greater canopy cover [39]. Our study region had two seasonal periods defined by precipitation patterns: the dry season (May to October, median maximum daily temperature: 28.8 °C, range: 16.8–40.7 °C) and the wet season (November to April, 31.1 °C, 19.9–42.1 °C, Fig. 1) [27].

### GPS data collection

From 2011 to 2018, we attached tracking collars, configured to collect GPS locations every five to sixty minutes, with sampling frequencies varying within deployments, to 24 wild dogs from 13 packs, 14 lions across seven social groups, and five cheetahs from different social groups (Fig. 1A-C; Figure S1) [40]. The collars were fitted in collaboration with a Botswana-registered veterinarian using species-specific immobilisation protocols (see [40–42]). Collar weights were always less than 2% of the bearer’s body mass (from 340-gram collars for cheetahs and wild dogs to 970 g for lions). We located collared animals every two to three weeks to remotely download collar data to a handheld terminal within field vehicles. All tracking collars were removed following battery expiry or upon completion of the study. We received approval for our work from the Royal Veterinary College Ethics & Welfare Committee and the Department of Wildlife and National Parks, Botswana.

Wild dogs are a cooperative species that move as a pack, and GPS data from a single individual can be used to represent group movements across the landscape [43–46]. As a result, we included tracking data from only one collared individual within each pack at any given time to avoid pseudoreplication. Moreover, we only included data from non-denning (non-young pup-rearing) wild dog packs due to unique patterns of space use during this period [14] and the limited amount of data to consider encounters during this period separately. In contrast, lions, which are also a social species, live in fission-fusion social structures, characterised by dynamic subgroup formations where individuals within a pride frequently split into smaller groups and join back together over time (though based on year-round field observations, we know such dynamics are common in our system, we lack empirical data on the frequency of sub-group formation) [47]. Thus, we calculated the distance between temporally simultaneous GPS fixes for each pair of lions within the same social group. For lion pairs that had more than 50% overlap in GPS locations, determined using a 400-meter threshold, indicating that they frequently travelled together, we randomly removed one individual from each pair from all subsequent analyses. This approach prevented issues of pseudoreplication arising from the non-independence of movement data within lion social groups. Cheetahs are largely solitary, and no coalitions of more than one individual were collared during our study; hence, the full dataset was used.

### Classification of temperature

We used the R package *mcera5* to download ERA5 reanalysis temperature data at the core of our study area (-19°52’S, 23°63’E) from 2011 to 2018 [48, 49]. The ERA5 reanalysis dataset is a widely used global gridded climate dataset provided by the European Centre for Medium-Range Weather Forecasts at a temporal resolution of one hour and a spatial resolution of 0.25° [48]. We collapsed hourly temperatures to derive daily maximum temperatures by taking the highest temperature for each calendar day. We used daily maximum temperature as we were interested in the spatial responses of animals across cooler and hotter days, and daily maximum temperature has been shown to be an important predictor of African large carnivore behaviour [1, 11, 50]. We scaled and centred maximum daily temperature covariates prior to inclusion within models to ease model interpretation [51].

### Modelling close encounters between dyads

We used the R package *wildlifeDI* to compare GPS data between individual lions and cheetahs and between individual lions and wild dogs, monitored concurrently, to determine the minimum daily distances between pairs of animals (dyads). We defined GPS fixes from dyad members as concurrent when they were sampled within 2.5 min of one another, which constitutes half of the highest sampling frequency, as recommended by [52]. We then measured distances between pairs of concurrent fixes. We used the full GPS data, as attempts to regularise the data to five-minute intervals resulted in unrealistic movement paths, while downsampling to hourly fixes would have reduced our ability to detect close encounters, which can unfold over only a few minutes (personal observation [53]). We excluded competitor dyads that overlapped temporally, but that were never recorded within 1,000 m of one another, i.e., dyads that occupied different regions of the study area and thus were unlikely to meet. To ensure our results were robust to the threshold used, we repeated our analyses with thresholds of 5,000 and 10,000 m. There were no differences in results, and we thus report model results from our 1,000 m exclusion threshold.

We defined close encounters, herein referred to as encounters, between dyad members as occurring on days when individuals were within at least 400 m of one another at the same time at some point in the day. This metric reflects close spatial proximity where dyad members may have been aware of one another, rather than a direct behavioural interaction. Nevertheless, our threshold was ecologically relevant as it was selected based on field experiments and observations on species detection distances within intermediately vegetated woodlands [54], and it follows thresholds established within similar systems [13, 55]. As such, this threshold represents an ecologically meaningful zone of proximity, rather than a behavioural cutoff. To ensure that our results were robust to our choice of threshold, we replicated our analyses with thresholds of 600, 800, and 1,000 m. It was not possible to repeat our analyses with smaller thresholds due to limited records at these values (e.g., cheetah-lion, threshold of 200 m: n_dry season_ = 17, n_wet_ = 6), which may reflect the true absence of these events or limited detections because such close encounters are fleeting (personal observation), sometimes unfolding over minutes, and thus may be missed by our sampling rate. No differences in results were found across thresholds; thus, we report the results of our 400 m threshold analyses.

We modelled the binary occurrence of encounters (1/0) as a function of maximum daily temperatures at both lag_0_ (current day) and lag_1_ (previous day) using generalised additive mixed models (GAMMs) [56]. We included maximum daily temperature at lag_0_ and lag_1_ to capture both immediate and delayed thermal responses. For example, immediate effects may influence activity timing or behaviour on the day of an encounter, whereas delayed effects may reflect changes in space use that require time to manifest as animals move across the landscape. Temperature covariates were included using thin-plate regression splines to allow for potential nonlinear relationships. We evaluated concurvity between smoothed lagged temperature terms using the *mgcv* package [56]. All diagnostics indicated moderate but non-problematic concurvity between lag_0_ and lag_1_ temperatures. Specifically, diagnostics showed concurvity coefficients under 0.8 and VIF coefficients under 10, i.e., under thresholds considered problematic for model interpretability and parameter estimate stability [57, 58]. As such, both lagged temperature terms were retained with the same models. We also incorporated a smooth spline term for the day of the year to control for seasonal variation in encounters driven by unmeasured environmental or behavioural factors. We included dyad identity as a random effect to account for repeated measurements within dyads. Additionally, we fitted an AR(1) autocorrelation structure to control for temporal autocorrelation within each dyad. Separate GAMMs were fit for each species (lion, cheetah, and wild dog) and each seasonal period (dry and wet seasons) to allow for species- and season-specific responses in encounters. All analyses were conducted using the *mgcv* package in R version 2.2.2 [56]. For all subsequent (non-encounter) analyses, we downsampled our GPS data to hourly intervals.

Upon running our analyses, we found that despite functional similarities, cheetahs and wild dogs showed divergent patterns of encounters with lions. To disentangle whether increased encounters were driven by changes in space use, temporal activity, or both, we then examined the influence of temperature on (i) interspecific spatial overlap, (ii) habitat selection, and (iii) diel activity overlap. As increased encounter probabilities were only observed during the dry season, we focus on dry season results in the main text; wet season analyses are provided in the supplemental material.

### Proportion of time within competitor home ranges

To assess changes in spatial overlaps across temperatures, we created rolling 30-day home ranges for each individual using Brownian bridge movement models, implemented via the kernel approach [59]. Specifically, for each individual and day of interest, we calculated the home range using GPS location data collected over the preceding 30-day period. Home ranges were generated only for periods with at least 24 days of location data available to ensure reliable home range estimation, and after regularising the GPS data to one location per hour, per previous studies in the system [13].

For each individual within each lion-cheetah and lion-wild dog dyad, we then calculated the proportion of hourly GPS fixes that fell within the home range of their competitor, using the competitor’s previous 30-day home range. This measure quantified the extent to which individuals spatially overlapped with their competitors. To investigate the influence of temperature on spatial overlap between competitors, we modelled the proportion of fixes within competitor home ranges using GAMMs with the same modelling structure as for encounters.

While both spatial overlap and encounter probability were derived from GPS data, they capture processes operating at different spatial and temporal scales. Spatial overlap reflects broader patterns of shared space use over 30-day periods, whereas encounters represent fine-scale, simultaneous proximity events.

### Modelling habitat selection

We used integrated step selection analyses (iSSA) to model habitat selection as a function of temperature in a use-availability framework [60]. As we were interested in habitat selection during movement, we subsampled GPS datasets to contain only movement locations, which we defined as consecutive GPS locations, resampled at hourly intervals, separated by at least 20 m. This movement threshold was selected based on the GPS collar error for fixes and aligns with thresholds used in previous work [11]. For each GPS location (herein referred to as a *used location*), we generated 150 paired available locations by simulating movement steps originating from the animal’s previous location. Our choice of the number of available locations was based on a sensitivity analysis of the impact of the number of available steps on coefficient estimates, as recommended by [61]. Available locations thus represented a set of alternative locations to each used location where the individual could have chosen to move. To do this, we fit gamma distributions to observed step lengths and von Mises Distributions to observed turning angles for each species, and we generated random step lengths and turning angles by sampling from those distributions [60]. For each location (used and available), we assigned habitat type, extracted from a raster (resolution 10 × 10 m) created from geo-referenced ortho-photographs (see [39]), and maximum daily temperature covariates corresponding to the day of interest and the preceding day. Specifically, each habitat type (grassland, floodplain, mixed forest, and mopane forest) was assigned to a binary variable, where 1 indicated presence in that habitat at the timestep. We expected species to select habitats differently across seasons; thus, each location was additionally classified as dry season or wet season.

For each species, we fit separate iSSA models for each season. Models took the exponential form, w(x) = exp(βx), with x a vector of covariates associated with each location and β a vector of associated coefficients, estimated via conditional logistic regression. We did not model habitat selection within a GAMM framework due to prohibitive computational times, and instead we modelled them within the *glmmTMB* package [62]. For each model, we related the relative probability of selection (w**(x)**) as a function of habitat type and temperature (Eq. 1). To test the hypothesis that temperature mediates habitat selection, we included maximum daily temperature, at lags 0 and 1, as interactions with habitat type terms to allow selection to vary across temperature gradients. Temperatures were not included as stand-alone fixed effects because they were the same for used and available locations for each step. We included day of the year in an m-spline with four degrees of freedom, following methods commonly used for modelling day of the year patterns (e.g. [63–65]), to account for unmeasured environmental variation. Additionally, to account for an underlying movement process independent of environmental factors, we included within models the step length, the log of the step length, and the cosine of the turning angle, *sensu* [66]. We used generalised estimating equations to estimate robust standard errors and treated each individual as a separate cluster [67].

1 $$\eqalign{& w(x) = \exp ({\beta _1}{\mkern 1mu} {\rm{grassland}} + {\beta _2}{\mkern 1mu} {\rm{grassland*temperature0}} \cr& + {\beta _3}{\mkern 1mu} {\rm{grassland}} * {\rm{temperature1}} + {\beta _4}{\mkern 1mu} {\rm{floodplain}} \cr& + {\beta _5}{\mkern 1mu} {\rm{floodplain}} * {\rm{temperature0}} + {\beta _6}{\mkern 1mu} {\rm{floodplain}}*{\rm{temperature1}} \cr& + {\beta _7}{\mkern 1mu} {\rm{mixed\: forest}} + {\beta _8}{\mkern 1mu} {\rm{mixed forest}}*{\rm{temperature0}} \cr& + {\beta _9}{\mkern 1mu} {\rm{mixed\: forest}} * {\rm{temperature1}} + {\beta _{10}}{\mkern 1mu} {\rm{step\: length}} \cr& + {\beta _{11}}{\mkern 1mu} \cos ({\rm{turning\: angle}})) + {\beta _{12}}{\mkern 1mu} {\rm{day\: of\: the\: year}} \cr} $$

### Temporal overlap

Time is another key dimension through which species can reduce their exposure to risk by shifting their activity to avoid peak periods of predator activity [68]. To assess whether temperature-driven changes in temporal overlap contributed to encounter risk, we extended previous analyses of temporal partitioning among African predators [11] to test whether temperature-driven changes in activity persisted into the following day. Specifically. we fit separate GAMMs for each species and season to assess how maximum daily temperatures influenced temporal partitioning within lion-competitor dyads, both on the day of (lag_0_) and the day following (lag_1_) temperatures. We first modelled species activity as a function of temperature. In line with activity delineations used within our iSSA, hourly animal activity was classified as either active or inactive based on a 20 m movement threshold between consecutive GPS fixes, also aligning with protocols from Rafiq et al., 2024 (see [11] for further details on data processing and sensitivity testing). These activity data were paired with ERA5-derived maximum daily temperature data and modelled using species- and season-specific GAMMs. Activity status (0/1) was the response variable. To control for seasonal shifts in sunrise and sunset times, we used *sun time*, a transformation of clock time that anchors sunrise and sunset at π/2 and 3π/2, respectively. Tensor interactions between sun time and maximum daily temperatures at lag_0_ and lag_1_ were included to model how daily activity patterns changed as a function of temperature. Models included a smooth term for day of the year to account for unexplained environmental variation, dyad identity as a random effect, and an AR(1) autocorrelation structure to account for temporal dependence within observations.

To assess whether activity overlaps between competitor dyads changed as a function of maximum daily temperatures, we created species activity predictions using our previous species activity models. We used predictions at the 20th and 80th temperature percentiles for each temperature lag, representing cool and warm days, to estimate the coefficients of overlap for each dyad (cheetah-lion and wild dog-lion) for each season. In other words, for each dyad, we looked at the overlap in activity timings for each species at cool temperatures and the overlap in activity timings for each species at warm temperatures. The coefficient of overlap measures the shared area between two kernel density estimates by taking the minimum value at each time point and integrating across the full period, and it ranges from 0 (no overlap) to 1 (complete overlap). We considered temperature to have a significant effect on overlap when the 95% confidence intervals for overlap estimates between the 20th and 80th temperature percentiles of the same temperature lag did not overlap.

Though maximum daily temperatures were included as continuous terms within models, to ease model interpretation when plotting or describing model results across temperatures, we often refer to cool, warm, and hot temperature days, using 20^th,^ 80^th,^ and 90th percentiles of maximum daily temperatures, consistent with thresholds commonly applied in climatological and ecological studies (e.g. [69, 70]).

## Results

Between 2011 and 2018, we tracked lions (*n* = 14), cheetahs (*n* = 5), and wild dogs (*n* = 24), covering an area of 2,600 km^2^ over a wide range of temperatures (range: 17–42 ° C) across both the dry and wet seasons.

### Cheetah-lion close encounters increase following warmer temperatures

**Fig. 2:**
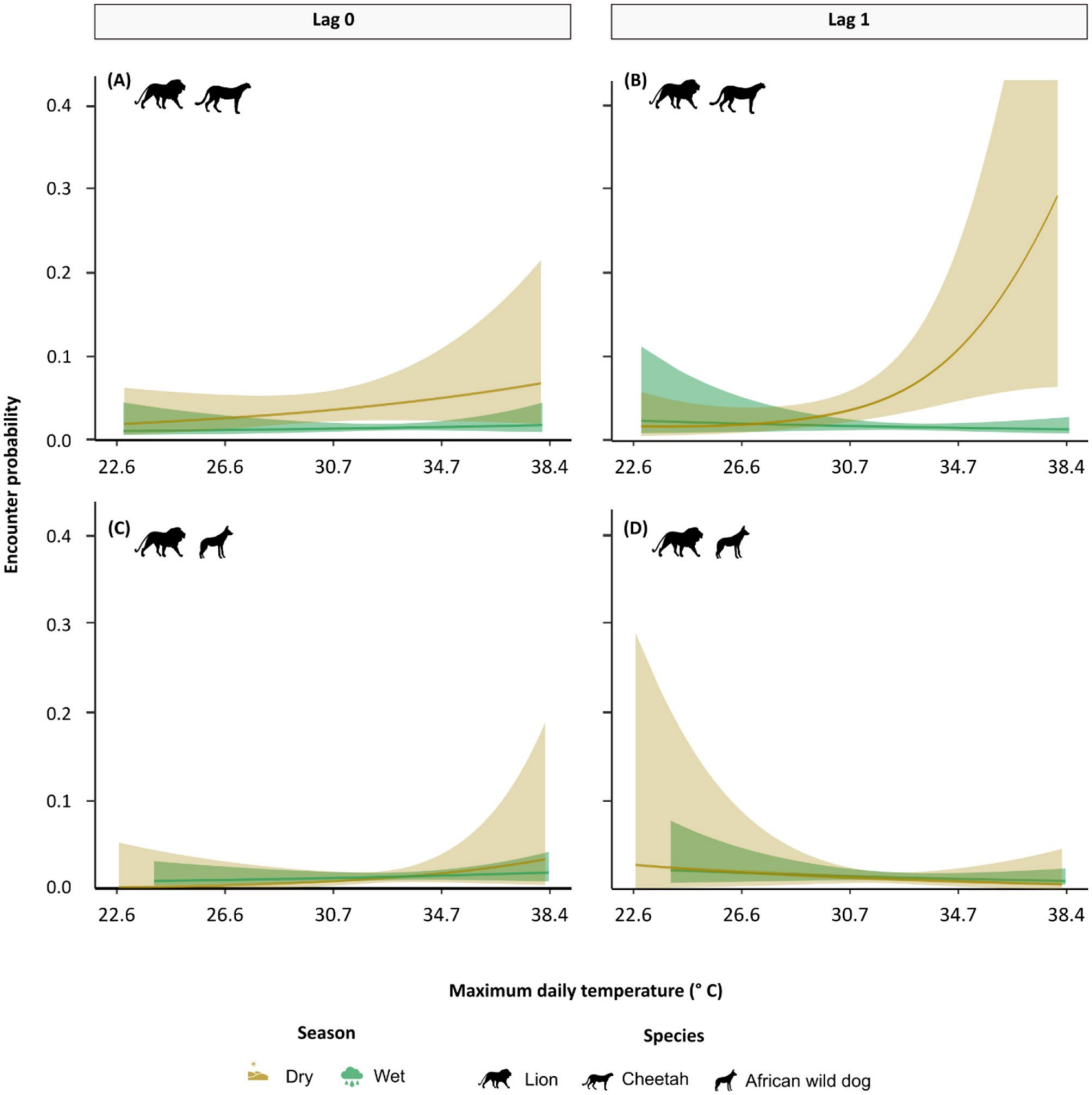
Cheetah-lion (**A**-**B**) and African wild dog-lion (**C**-**D**) encounter probabilities across maximum daily temperature ranges on the day of encounters (lag_0_) and the day before encounters (lag_1_). Maximum daily temperature tick marks correspond approximately to -2, -1, 0, + 1, and + 2 standard deviations from the mean. Shaded ribbons represent 95% confidence intervals and for panel B are clipped at 0.4 for readability (confidence interval reaches 0.78)

We used generalised additive mixed models to quantify whether close encounters, defined as events when animals were within 400 m of one another [13], between cheetahs and lions and between wild dogs and lions increased as a function of maximum daily temperatures on the day of the encounter (lag_0_) or the day prior (lag_1_). We included lagged temperatures to account for the time animals require to move across the landscape in response to thermal conditions. During the dry season, warmer temperatures on the day prior (lag_1_) significantly increased the likelihood of cheetah-lion encounters (R^2^ = 0.03, F-statistic = 4.211, p-value = 0.014). This relationship was non-linear, with encounter probabilities remaining consistently low at temperatures below the study period median (Fig. 2). However, once temperatures surpassed the median, encounter probabilities rose rapidly, with encounters 3.52 and 6.64 times more likely following warm (80th percentile) and hot (90th percentile) days, respectively, compared to median temperatures. In contrast, temperature did not influence encounter probabilities between cheetahs and lions during the wet season, or between wild dogs and lions in either season (Fig. 2; Table S1).

### Cheetahs and lions increase spatial overlap following warmer temperatures

**Fig. 3:**
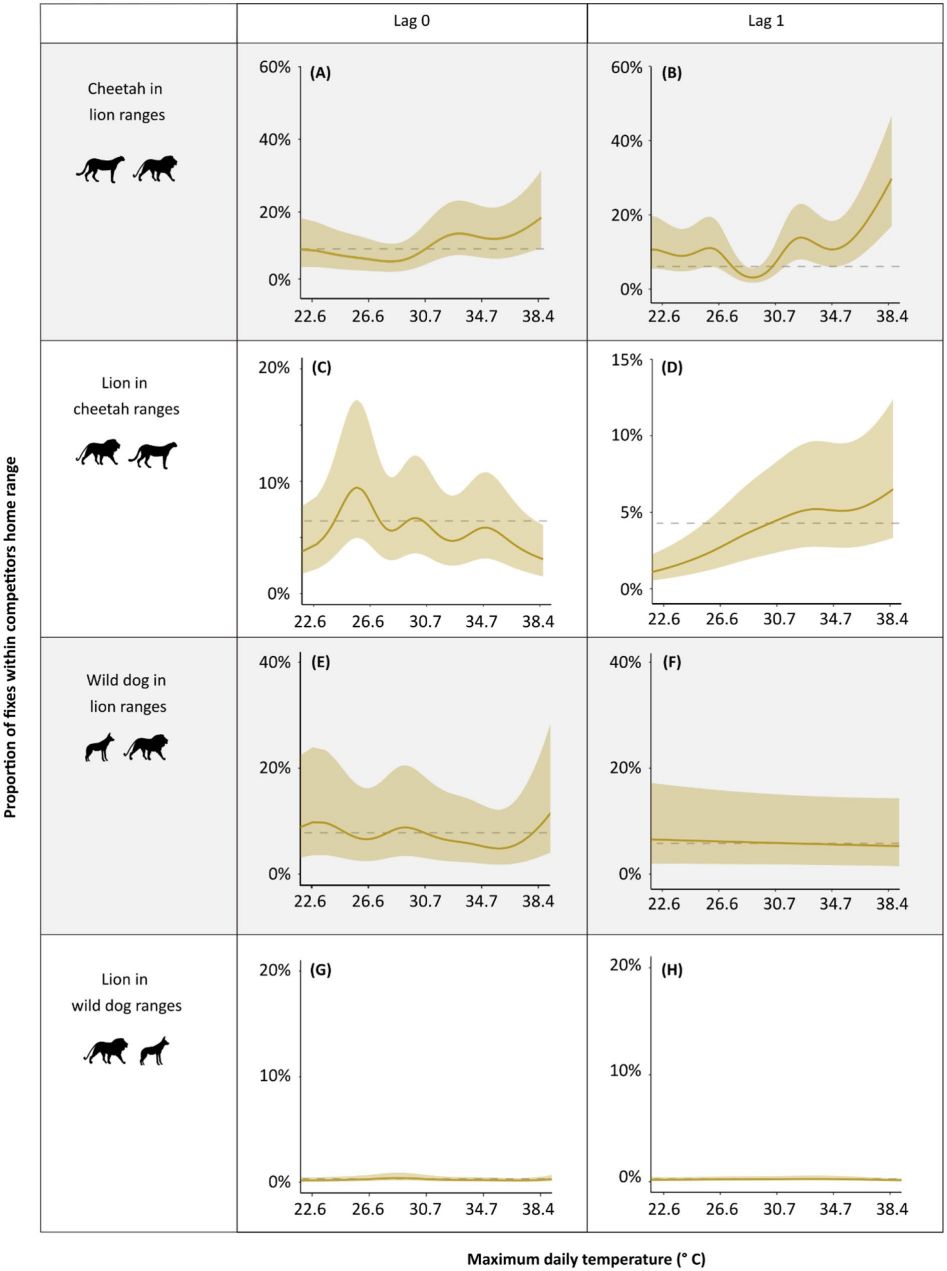
Proportion of GPS locations within competitor home ranges in relation to maximum daily temperatures on the day of (lag_0_) and the day prior (lag_1_). Horizontal dashed lines indicate the proportion of locations within competitor home ranges at median temperature values. Maximum daily temperature tick marks correspond approximately to -2, -1, 0, + 1, and + 2 standard deviations from the mean. Shaded ribbons represent 95% confidence intervals

Cheetahs and lions spent more time (measured as the proportion of GPS fixes) within each other’s home ranges on the day following warmer temperatures, relative to median temperatures. Cheetah presence in lion ranges increased by 3.55-fold the day following warm days (80th percentile) and more than quadrupled (4.22-fold increase) after hot days (90th percentile), compared to median temperatures (R² = 0.02, F = 32.15, *p* < 0.001, Fig. 3A-B, Table S2). Lions showed a similar, though less pronounced, increase in their presence within cheetah home ranges, with a 1.39-fold increase in time spent within cheetah ranges the day following warm and hot temperatures (R² = 0.02, F = 17.93, *p* < 0.001, Fig. 3C-D, Table S2). In contrast, wild dog spatial overlap with lions remained consistent across all temperature ranges (Fig. 3E-H, Table S2), paralleling the same patterns observed in temperature-mediated encounter probabilities.

### Cheetahs increase their selection of high-risk lion areas following warmer temperatures

**Fig. 4:**
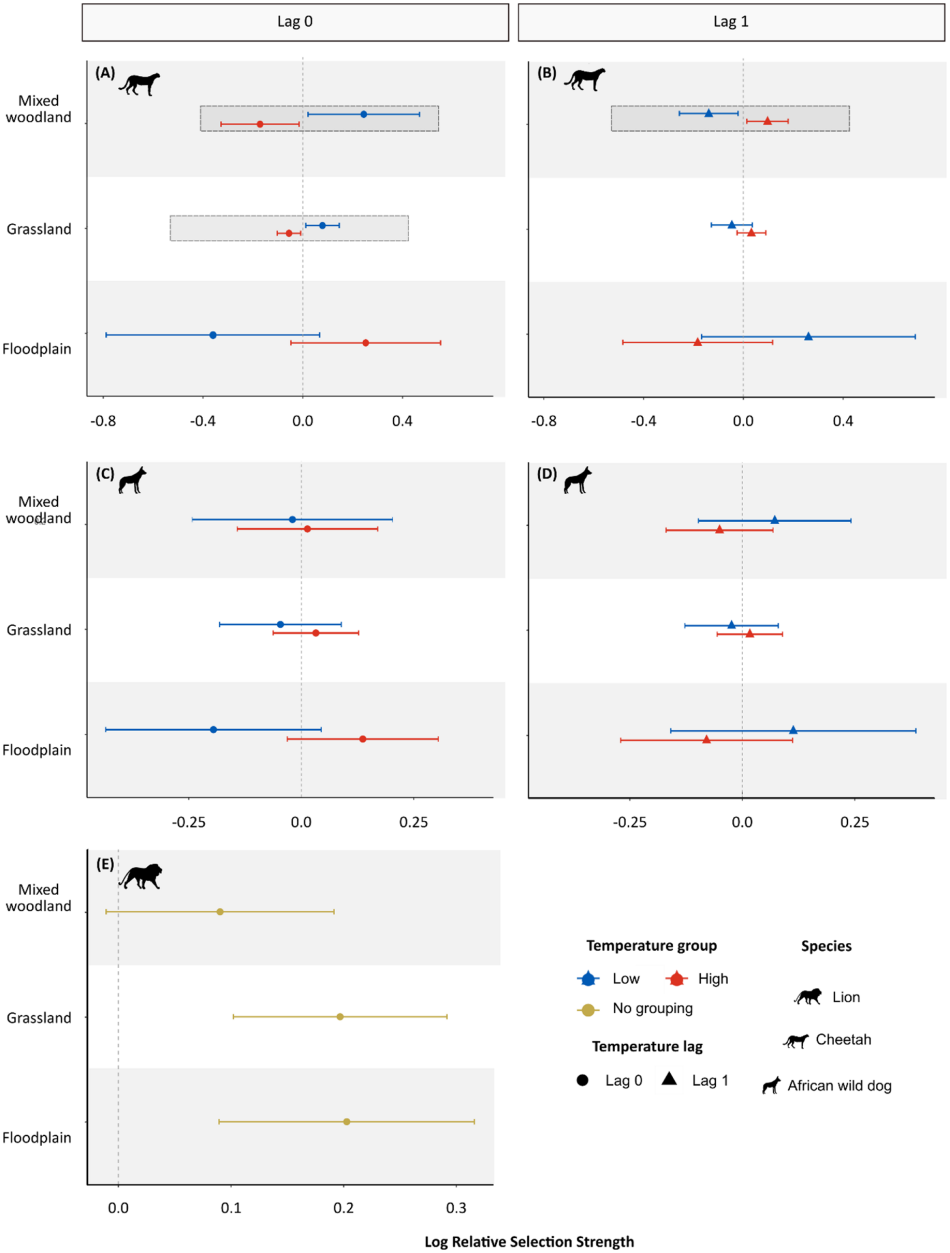
Relative selection strength estimates for mixed woodlands, grasslands, and floodplains, relative to mopane woodlands, across cool (20th percentile) and warm (80th percentile) maximum daily temperatures and across time lags for each predator species. Dashed vertical lines represent no change in selection. Positive values indicate selection for that habitat and negative values represent avoidance. Error bars represent 95% confidence intervals. Dashed boxes indicate habitats for which there was a statistically significant change in selection as a function of temperature

Paralleling increases in encounters, cheetah selection for moderately densely vegetated habitats (mixed woodlands) increased in the day following warm temperatures, whilst wild dog habitat selection did not change across temperatures (Fig. 4, Table S3-4). Interestingly, the reverse pattern occurred on warm days themselves, with cheetahs avoiding mixed woodlands, suggesting a complex, time-lagged response to thermal conditions (Tables S3). Though lion habitat selection remained unchanged across temperature gradients, lions showed general selection for floodplains and grasslands and no selection or avoidance of mixed woodlands, irrespective of temperature, relative to mopane woodlands (Table S5).

### Warm days reduce temporal partitioning between cheetahs and lions

**Fig. 5:**
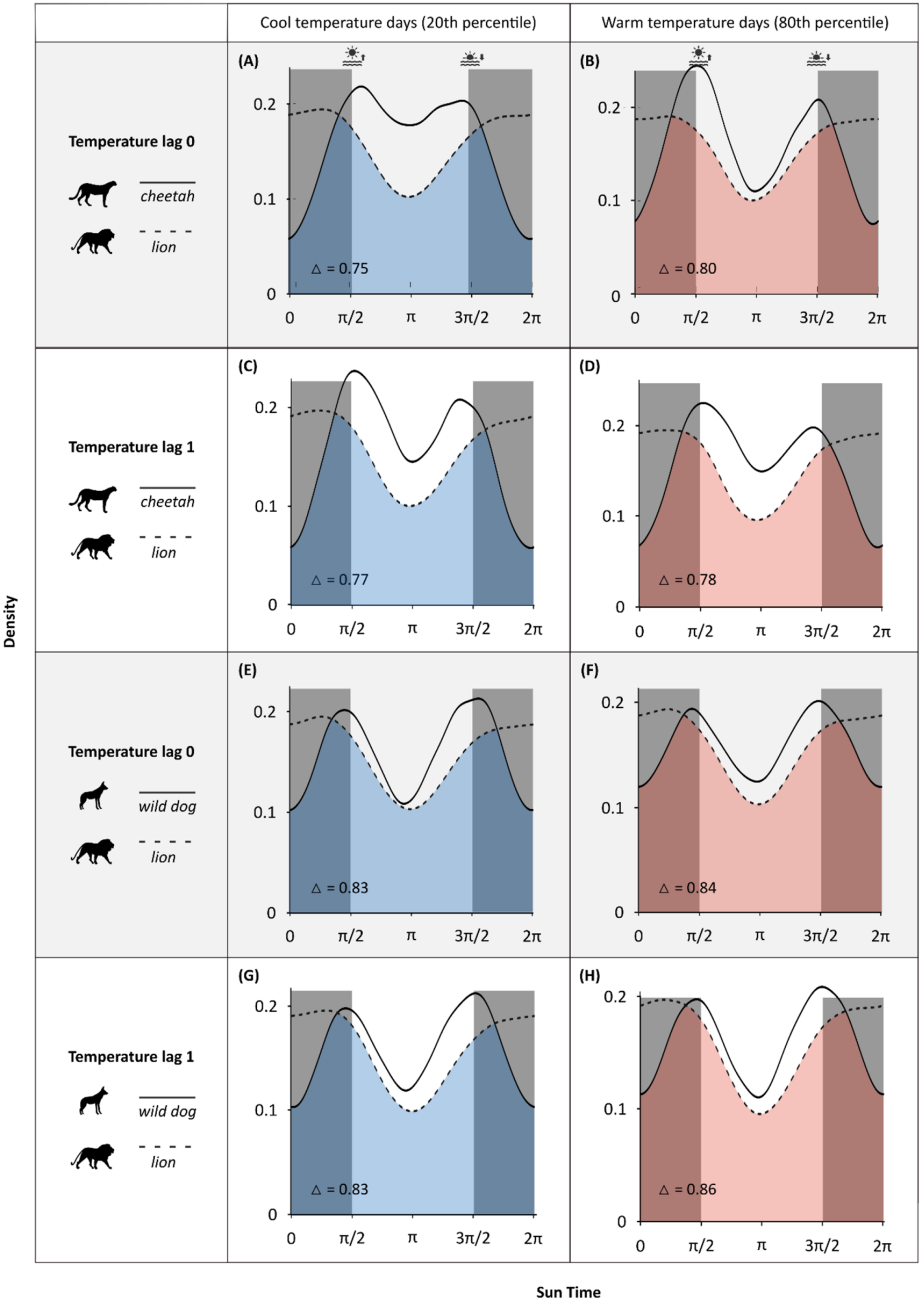
Changes in temporal partitioning between cheetahs and lions and between African wild dogs and lions across cool (20th percentile) and warm (80th percentile) maximum daily temperatures and across temperature lags. Curves denote species kernel densities for activity. Shaded and unshaded panels on each plot delineate night and day, respectively, with the transitions between the two representing sunrise and sunset. △ denotes the coefficient of activity overlap (where 0 indicates no overlap and 1 indicates full overlap)

We found that cheetahs became more nocturnal during warmer conditions (R² = 0.15, F = 54.28, *p* < 0.001), increasing their temporal overlap with lions, whose activity remained largely nocturnal and stable across temperature gradients (Table S6). We found no evidence that this shift toward nocturnality persisted into the day after hot temperatures (Fig. 5, Table S6). As a result, cheetah–lion activity overlaps marginally increased on warmer days, with a 7.47% rise in overlap on warm (80th percentile) compared to cool (20th percentile) temperatures, based on non-overlapping confidence intervals (Table S7), and showed no difference on the day following warmer temperatures. There was no significant change in activity overlaps between wild dogs and lions due to minimal changes in activity timings across temperatures for either species (Table S6-7).

## Discussion

Given the rapid pace of global environmental change, understanding how climate variability reshapes animal-environment relationships and ecological interactions is increasingly urgent [71]. How animals adjust their space use in response to climate can influence ecological processes across multiple scales, from individual fitness [72] to population dynamics [73] and community interactions [74]. In this study, we show that increases in ambient temperatures significantly increase the likelihood of close encounters, herein encounters, and spatiotemporal overlap between two apex predators – cheetahs and lions – providing empirical evidence linking climate variability to changes in encounter rates among top predators. In contrast, temperature had no impact on lion–wild dog interactions, consistent with previous evidence that wild dogs show strong proactive avoidance of lions across a range of environmental contexts [75–77]. Shifts in species interactions are a key pathway through which climate variability can threaten population persistence [15–17]. Our finding that encounters between cheetahs and lions increased under warmer temperatures represents one of the first empirical examples of changes in encounters between top endothermic predators due to temperature variability. As lions are competitively dominant over cheetahs and impose kleptoparasitism, injury, and mortality risks [34–36], increased encounters under warming temperatures could elevate predation or competition risks for cheetahs, which could have consequences for population dynamics, though we acknowledge that not all close encounters may result in antagonistic interactions, and we lacked data to test their consequences for population dynamics within our study. For example, it remains possible that cheetahs may tolerate closer proximity to lions during warmer conditions if reductions in lion movement and activity limit the likelihood of antagonistic interactions.

The changes in predator encounters we observed corresponded closely with lagged patterns of spatial overlap, with cheetahs showing greater shifts in overlap with lions during warmer periods than did wild dogs, suggesting that broad-scale changes in space use may have contributed to temperature-mediated increases in encounters. Several mechanisms could contribute to these spatial responses. For example, according to the heat dissipation hypothesis, an animal’s ability to shed excess heat fundamentally constrains its behaviour and may drive increased interspecific spatial overlaps [79, 80]. Thus, an untested hypothesis is that increased encounters during warmer, drier temperatures may reflect a potential trade-off between, for example, accessing stable water sources and shade (which are both important for evaporative and radiant cooling) and avoiding encounters with dominant competitors. In our system, warmer and drier periods may therefore draw individuals toward stable water sources or intermediate-vegetated habitats that offer shade, even though during dry periods, lions aggregate in these areas due to prey concentrations [81, 82]. Indeed, previous research in our system has shown that drought conditions increase spatial overlap between large predators [13], highlighting the important role water availability plays in shaping community dynamics. Another plausible, non-exclusive explanation is that temperature-mediated changes in prey distribution may indirectly mediate predator movements. Indeed, warming temperatures are known to increase African herbivore aggregation [79], potentially affecting predator movements and encounter probabilities. Encounter patterns may also reflect changing habitat type preferences across temperatures. Lions are ambush predators, with hunting success increasing within intermediate vegetated woodlands [83]. Mixed woodlands in our study area may thus represent zones of elevated encounter risk due to reduced visibility. Thus, their increased selection by cheetahs in the days following warmer temperatures, which may reflect shifting shade, prey or other important resource distributions, may contribute to the increased encounter probabilities observed. Under our initial hypothesis of increased selection for shaded habitats under warmer temperatures, we would expect increased selection of woodlands on the day of warm temperatures; however, the time-lagged response of selecting woodland areas the day after warm temperatures suggests that more than shade availability is driving these patterns. Future work incorporating high-resolution microclimate data, dynamic maps of prey and competitor distributions, and fine-scale behavioural data would help clarify how these factors interact to shape predator behaviour and interspecific encounters. Interestingly, temporal changes in overlap between cheetahs and lions did not mirror encounter patterns, with temperature effects occurring on the day of (see also [11]), and having no impact on activity overlaps the day following, warmer temperatures, whilst spatial responses were lagged. This suggests that changes in spatial overlap may be the primary driver, over temporal partitioning, for temperature-mediated increases in encounter probabilities between cheetahs and lions.

Interestingly, despite lions also being a major source of mortality for wild dogs [38, 84], we found no evidence of a change in encounters and overlaps between the species across temperatures. This could indicate that strong, proactive lion avoidance by wild dogs persists across temperature gradients. However, this does not imply broader resilience to climate change, as wild dogs remain vulnerable to other heat-related constraints on activity and reproduction [11, 22, 50]. This aligns with emerging evidence suggesting that wild dogs are more sensitive to population suppression from lions than cheetahs [38], and often prioritise proactive lion avoidance against other competing costs, such as selecting for areas with lower lion densities even when there is higher anthropogenic risk [76], lower prey availability [14, 75], and reduced water accessibility. These results highlight the markedly different role climate variability can play in mediating close encounters of two functionally similar species with the same top predator (which we discuss further later). Although our movement-based analyses cannot distinguish among the proximate drivers of these changes, several plausible mechanisms, including shifts in prey distribution, water access, and thermal constraints, may combine to shape how predators make behavioural decisions across temperature gradients.

The diversity of species responses we observed to temperature may reflect species-specific ecological and evolutionary drivers shaping movements, such as risk landscapes and foraging constraints [85–87]. Cheetahs typically exhibit reactive avoidance to lion risk, adjusting their behaviours primarily when in close proximity to lions [27, 88], whereas wild dogs proactively avoid high-risk lion habitats [26, 89]. Moreover, cheetahs are more diurnal, relative to the crepuscular activity of wild dogs. Thus, the greater exposure of cheetahs to higher daytime temperatures, coupled with their relatively close proximity to lions, may necessitate them carrying out heat-mitigation behaviours even under conditions where it elevates their risk to lion encounters. In contrast, wild dogs, which are primarily active during cooler periods and already avoid lions at large spatial scales [11, 75, 76], may experience less physiological or ecological pressures to modify behaviour on warmer days, and even when they are required to, may have greater flexibility to engage in such behaviours without increasing their risk of lion encounters, due to their already greater spatial segregation from lions. Thus, such spatial responses to temperature appear to interact with, rather than replace, the established avoidance strategies of these species. In other words, wild dogs’ strong baseline avoidance of lions may buffer them from temperature-related changes in risk. While our GPS-based approach cannot directly test the specific behavioural mechanisms at play, this interpretation aligns with established differences in daily activity patterns, risk avoidance strategies, and thermal exposure among these species [11, 25, 27, 38, 46]. Similar proactive avoidance of competitor risk is seen within other predator assemblages, such as between coyotes (*Canis latrans*) and mountain lions (*Puma concolor*) in North America [90], and between leopards (*Panthera pardus)*, tigers (*Panthera tigris*), and dholes (*Cuon alpinus*) in Asia [91]. An interesting arising question is whether temperature-driven changes in encounters may be generally mediated by proactive versus reactive risk avoidance strategies employed by subordinate predators, for example with species that employ reactive avoidance strategies perhaps being more tolerant to undertaking riskier behaviours. Additionally, our finding that lions did not alter their habitat selection across temperatures contrasts with predictions from allometric theory [92–94] that larger-bodied species should be more thermally sensitive, but this may be explained by their sit-and-ambush hunting strategy, which favours vegetated habitats providing camouflage and thermal cover [83] and which minimises the risks of hyperthermia during hunts [80]. While the specific pathways through which temperature impacts movement in our system, for example thermal tolerance versus prey tracking, remain unknown due to limited data on prey distributions and microclimates during our study, our findings offer testable hypotheses for future research incorporating spatially explicit prey and resource availability data.

That interspecific encounters increase, and spatiotemporal partitioning decreases, under warmer temperatures is concerning in light of ongoing climate change. While temperature variability is natural, the frequency and persistence of hot days in many systems, including those within southern Africa, are increasing due to anthropogenic climate change [33, 95], and our results suggest these conditions may draw some species into closer contact more often than in the past. Since the late 1980s, maximum daily temperatures within the Okavango Delta, Botswana, have risen on average by approximately 1.6 °C [1]. Climate projections suggest this trend will continue, with increases in overall temperatures, frequency of hot days, and severity of heatwaves expected over the next several decades [33]. Meanwhile, key thermoregulatory resources like water and shade are likely to become scarcer due to intensifying drought and aridification. Such shifts could exacerbate competition and encounters among predator species, compounding already existing challenges related to thermal stress and resource scarcity. It is also worth noting that beyond the Okavango Delta, these species are often increasingly restricted to smaller reserves, where opportunities for spatial avoidance may be limited by habitat loss, fragmentation, and fencing. In these restricted landscapes, temperature-driven spatial shifts may disproportionately amplify interspecific interactions, intensifying the ecological consequences of competition.

## Conclusion

Our results provide one of the first examples of temperature variability driving changes in close encounters in top-endothermic predators. As ecosystems undergo rapid environmental change, key resources for thermoregulation (such as water and shade) change in their availability and distribution, and carnivore ranges become increasingly fragmented, understanding how climate change alters encounters between species is increasingly critical. Moving forward, integrating behavioural, demographic, and climatic data will be essential for predicting how warming reshapes the structure, persistence, and resilience of predator guilds in rapidly changing landscapes.

## Electronic Supplementary Material

Below is the link to the electronic supplementary material.

Supplementary Material 1

## Data Availability

The datasets and code supporting the conclusions of this article are available at 10.5281/zenodo.19127838.
