# Supplementary material for: Warming temperatures increase close encounters between two top predator species via changes in spatial behaviour
Source: Mov Ecol. 2026 Apr 11;14:32. doi: 10.1186/s40462-026-00635-z (PMC13182089; doi:10.1186/s40462-026-00635-z)
Supplement: Supplementary file 1 — Supplementary Material 1 [file 40462_2026_635_MOESM1_ESM.docx]

**Supplemental materials**

**Wet season results**

We found little evidence of temperature-mediated changes in spatial overlap between cheetahs and lions and between African wild dogs and lions in the wet season. Specifically, the proportion of time species spent within competitor home ranges showed minimal changes in biologically relevant scales across temperatures in the wet season (Table S2) (Figure S2), and there were minimal temperature-mediated changes in habitat selection, with only wild dogs increasing their selection for floodplains under warmer temperatures (Tables S3-5) (Figure S3). Similar to the dry season, however, we found that cheetahs became increasingly nocturnal during warmer temperatures in the wet season, and this led to reduced temporal partitioning on warm days (Tables S6-7) (Figure S4).

**Table S1. Summary of generalised additive mixed models for analyses investigating close encounters within cheetah-lion and African wild dog-lion dyads.**

|  |  |  |  |  |  |  |  |
| --- | --- | --- | --- | --- | --- | --- | --- |
| **Term** | **Estimate** | **EDF** | **SE** | **Ref. df** | ***t*-value** | ***F*-value** | ***P*-value** |
| CHEETAH-LION (DRY SEASON) |  |  |  |  |  |  |  |
| (Intercept) | -4.595 |  | 0.234 |  | -19.646 |  | < 0.001 |
| s(lag0_temperature_max_scale) | | 1.000 |  | 1.000 |  | 1.241 | 0.265 |
| s(lag1_temperature_max_scale) | | 2.049 |  | 2.049 |  | 4.211 | 0.014 |
| s(day) |  | 3.375 |  | 3.375 |  | 8.471 | < 0.001 |
|  |  |  |  |  |  |  |  |
| CHEETAH-LION (WET SEASON) | |  |  |  |  |  |  |
| (Intercept) | -4.643 |  | 0.202 |  | -22.931 |  | < 0.001 |
| s(lag0_temperature_max_scale) | | 1.000 |  | 1.000 |  | 0.315 | 0.575 |
| s(lag1_temperature_max_scale) | | 1.000 |  | 1.000 |  | 0.396 | 0.529 |
| s(day) |  | 1.000 |  | 1.000 |  | 0.992 | 0.319 |
|  |  |  |  |  |  |  |  |
| WILD DOG-LION (DRY SEASON) | |  |  |  |  |  |  |
| (Intercept) | -4.375 |  | 0.254 |  | -17.246 |  | < 0.001 |
| s(lag0_temperature_max_scale) | | 1.000 |  | 1.000 |  | 1.115 | 0.291 |
| s(lag1_temperature_max_scale) | | 1.000 |  | 1.000 |  | 0.391 | 0.532 |
| s(day) |  | 1.000 |  | 1.000 |  | 0.037 | 0.848 |
|  |  |  |  |  |  |  |  |
| WILD DOG-LION (WET SEASON) | |  |  |  |  |  |  |
| (Intercept) | -4.493 |  | 0.203 |  | -22.186 |  | < 0 .001 |
| s(lag0_temperature_max_scale) | | 1.000 |  | 1.000 |  | 0.531 | 0.466 |
| s(lag1_temperature_max_scale) | | 1.000 |  | 1.000 |  | 0.605 | 0.437 |
| s(day) |  | 1.000 |  | 1.000 |  | 0.014 | 0.906 |
|  |  |  |  |  |  |  |  |
|  |  |  |  |  |  |  |  |
| EDF = estimated degrees of freedom, SE = standard error, Ref. df = reference degrees of freedom | | | | | | | |

**Table S2. Summary of generalised additive mixed models for analyses investigating time spent within competitor home ranges for cheetah-lion and African wild dog-lion dyads.**

|  |  |  |  |  |  |  |  |
| --- | --- | --- | --- | --- | --- | --- | --- |
| **Term** | **Estimate** | **EDF** | **SE** | **Ref. df** | ***t*-value** | ***F*-value** | ***P*-value** |
| Cheetahs in lion ranges (dry season) |  |  |  |  |  |  |  |
| (Intercept) | -3.892 |  | 0.300 |  | -12.980 |  | < 0.001 |
| s(lag0_temperature_max_scale) |  | 6.885 |  | 6.885 |  | 10.909 | < 0.001 |
| s(lag1_temperature_max_scale) |  | 8.652 |  | 8.652 |  | 32.145 | < 0.001 |
| s(day) |  | 8.864 |  | 8.864 |  | 70.133 | < 0.001 |
|  |  |  |  |  |  |  |  |
| Cheetahs in lion ranges (wet season) |  |  |  |  |  |  |  |
| (Intercept) | -3.349 |  | 0.299 |  | -11.200 |  | < 0.001 |
| s(lag0_temperature_max_scale) |  | 8.133 |  | 8.133 |  | 3.865 | < 0.001 |
| s(lag1_temperature_max_scale) |  | 2.161 |  | 2.161 |  | 1.362 | 0.189 |
| s(day) |  | 8.188 |  | 8.188 |  | 40.532 | < 0.001 |
|  |  |  |  |  |  |  |  |
| Lions in cheetah ranges (dry season) |  |  |  |  |  |  |  |
| (Intercept) | -3.042 |  | 0.332 |  | -9.166 |  | < 0.001 |
| s(lag0_temperature_max_scale) |  | 8.371 |  | 8.371 |  | 9.365 | < 0.001 |
| s(lag1_temperature_max_scale) |  | 4.672 |  | 4.672 |  | 19.552 | < 0.001 |
| s(day) |  | 8.385 |  | 8.385 |  | 33.722 | < 0.001 |
|  |  |  |  |  |  |  |  |
| Lions in cheetah ranges (wet season) |  |  |  |  |  |  |  |
| (Intercept) | -3.411 |  | 0.588 |  | -5.798 |  | < 0.001 |
| s(lag0_temperature_max_scale) |  | 2.788 |  | 2.788 |  | 12.620 | < 0.001 |
| s(lag1_temperature_max_scale) |  | 7.927 |  | 7.927 |  | 12.419 | < 0.001 |
| s(day) |  | 8.169 |  | 8.169 |  | 78.162 | < 0.001 |
|  |  |  |  |  |  |  |  |
| Wild dogs in lion ranges (dry season) |  |  |  |  |  |  |  |
| (Intercept) | -3.692 |  | 0.477 |  | -7.733 |  | < 0.001 |
| s(lag0_temperature_max_scale) |  | 7.090 |  | 7.090 |  | 5.279 | < 0.001 |
| s(lag1_temperature_max_scale) |  | 1.000 |  | 1.000 |  | 0.445 | 0.505 |
| s(day) |  | 8.664 |  | 8.664 |  | 23.065 | < 0.001 |
|  |  |  |  |  |  |  |  |
|  |  |  |  |  |  |  |  |
| Wild dogs in lion ranges (wet season) |  |  |  |  |  |  |  |
| (Intercept) | -3.325 |  | 0.239 |  | -13.903 |  | < 0.001 |
| s(lag0_temperature_max_scale) |  | 7.469 |  | 7.469 |  | 9.432 | < 0.001 |
| s(lag1_temperature_max_scale) |  | 7.879 |  | 7.879 |  | 8.059 | < 0.001 |
| s(day) |  | 8.082 |  | 8.082 |  | 23.826 | < 0.001 |
|  |  |  |  |  |  |  |  |
| Lions in wild dog ranges (dry season) |  |  |  |  |  |  |  |
| (Intercept) | -3.236 |  | 0.360 |  | -8.986 |  | < 0.001 |
| s(lag0_temperature_max_scale) |  | 6.725 |  | 6.725 |  | 5.558 | < 0.001 |
| s(lag1_temperature_max_scale) |  | 4.909 |  | 4.909 |  | 5.166 | < 0.001 |
| s(day) |  | 8.641 |  | 8.641 |  | 75.076 | < 0.001 |
|  |  |  |  |  |  |  |  |
| Lions in wild dog ranges (wet season) |  |  |  |  |  |  |  |
| (Intercept) | -2.211 |  | 0.316 |  | -6.990 |  | < 0.001 |
| s(lag0_temperature_max_scale) |  | 7.315 |  | 7.315 |  | 11.477 | < 0.001 |
| s(lag1_temperature_max_scale) |  | 8.414 |  | 8.414 |  | 14.170 | < 0.001 |
| s(day) |  | 8.780 |  | 8.780 |  | 78.403 | < 0.001 |
|  |  |  |  |  |  |  |  |
|  |  |  |  |  |  |  |  |
| EDF = estimated degrees of freedom, SE = standard error, Ref. df = reference degrees of freedom | | | | | | | |

**Table S3. Coefficient estimates of cheetah iSSA models.**

|  |  |  |  |  |  |  |  |  |
| --- | --- | --- | --- | --- | --- | --- | --- | --- |
| **Model term** | **Coefficient** | **Exp. coef.** | **SE** | **RSE** | **z-score** | **p-value** | **Lower 95% CI** | **Upper 95% CI** |
|  |  |  |  |  |  |  |  |  |
| DRY SEASON |  |  |  |  |  |  |  |  |
| Grassland | 0.726 | 2.067 | 0.076 | 0.074 | 9.821 | < 0.001 | 1.788 | 2.389 |
| Floodplain | 0.498 | 1.645 | 0.103 | 0.105 | 4.732 | < 0.001 | 1.338 | 2.022 |
| Mixed woodland | -0.052 | 0.949 | 0.099 | 0.092 | -0.568 | 0.570 | 0.793 | 1.137 |
| Day (spline 1) | 0.568 | 1.766 | 25.935 | 5.915 | 0.096 | 0.923 | 1.63E-05 | 1.91E + 05 |
| Day (spline 2) | -6.473 | 0.002 | 15.259 | 2.813 | -2.301 | 0.021 | 6.23E-06 | 0.383 |
| Day (spline 3) | 1.982 | 7.260 | 22.472 | 8.245 | 0.240 | 0.810 | 6.96E-07 | 7.58E + 07 |
| Day (spline 4) | -3.199 | 0.041 | 9.120 | 1.483 | -2.157 | 0.031 | 0.002 | 0.747 |
| Step length | -1.14E-04 | 1.000 | 4.16E-05 | 3.29E-05 | -3.481 | 0.001 | 0.9998 | 0.99995 |
| log (step length) | 0.331 | 1.392 | 0.039 | 0.041 | 7.972 | < 0.001 | 1.283 | 1.509 |
| cos (turning angle) | 0.045 | 1.046 | 0.044 | 0.012 | 3.832 | < 0.001 | 1.022 | 1.071 |
| Grassland * temperature (lag 0) | -0.067 | 0.935 | 0.090 | 0.029 | -2.305 | 0.021 | 0.883 | 0.990 |
| Floodplain * temperature (lag 0) | 0.308 | 1.360 | 0.178 | 0.187 | 1.650 | 0.099 | 0.944 | 1.961 |
| Mixed woodland * temperature (lag 0) | -0.209 | 0.811 | 0.147 | 0.097 | -2.147 | 0.032 | 0.671 | 0.982 |
| Grassland * temperature (lag 1) | 0.039 | 1.040 | 0.079 | 0.036 | 1.100 | 0.271 | 0.970 | 1.116 |
| Floodplain * temperature (lag 1) | -0.223 | 0.800 | 0.162 | 0.187 | -1.195 | 0.232 | 0.555 | 1.154 |
| Mixed woodland * temperature (lag 1) | 0.118 | 1.126 | 0.135 | 0.051 | 2.306 | 0.021 | 1.018 | 1.245 |
|  |  |  |  |  |  |  |  |  |
|  |  |  |  |  |  |  |  |  |
| WET SEASON |  |  |  |  |  |  |  |  |
| Grassland | 0.658 | 1.932 | 0.087 | 0.065 | 10.113 | < 0.001 | 1.700 | 2.195 |
| Floodplain | 0.690 | 1.994 | 0.162 | 0.097 | 7.126 | < 0.001 | 1.649 | 2.411 |
| Mixed woodland | 1.20E-04 | 1.000 | 0.113 | 0.025 | 0.005 | 0.996 | 0.952 | 1.051 |
| Day (spline 1) | 43.170 | 5.61E + 18 | 72.551 | 11.269 | 3.831 | < 0.001 | 1.43E + 09 | 2.19E + 28 |
| Day (spline 2) | -118.534 | 3.32E-52 | 127.634 | 9.181 | -12.911 | < 0.001 | 5.09E-60 | 2.17E-44 |
| Day (spline 3) | -3.050 | 0.047 | 41.970 | 7.695 | -0.396 | 0.692 | 1.34E-08 | 1.68E + 05 |
| Day (spline 4) | 3.483 | 32.547 | 7.399 | 1.542 | 2.258 | 0.024 | 1.584 | 668.669 |
| Step length | -2.18E-05 | 1.000 | 3.12E-05 | 1.39E-05 | -1.564 | 0.118 | 1.000 | 1.000 |
| log (step length) | 0.257 | 1.293 | 0.035 | 0.025 | 10.311 | < 0.001 | 1.232 | 1.358 |
| cos (turning angle) | 0.029 | 1.030 | 0.044 | 0.005 | 5.749 | < 0.001 | 1.020 | 1.040 |
| Grassland * temperature (lag 0) | 0.082 | 1.085 | 0.094 | 0.044 | 1.853 | 0.064 | 0.995 | 1.183 |
| Floodplain * temperature (lag 0) | 0.294 | 1.341 | 0.190 | 0.155 | 1.898 | 0.058 | 0.990 | 1.817 |
| Mixed woodland * temperature (lag 0) | 0.099 | 1.104 | 0.146 | 0.120 | 0.824 | 0.410 | 0.872 | 1.398 |
| Grassland * temperature (lag 1) | 0.061 | 1.063 | 0.094 | 0.059 | 1.033 | 0.302 | 0.947 | 1.192 |
| Floodplain * temperature (lag 1) | -0.117 | 0.889 | 0.190 | 0.126 | -0.926 | 0.354 | 0.694 | 1.140 |
| Mixed woodland * temperature (lag 1) | -0.032 | 0.969 | 0.145 | 0.044 | -0.726 | 0.468 | 0.889 | 1.055 |
|  |  |  |  |  |  |  |  |  |
|  |  |  |  |  |  |  |  |  |
|  |  |  |  |  |  |  |  |  |
| Exp. Coef = exponentiated coefficient; SE = standard error; RSE = robust standard error; CI = confidence interval | | | | | | |  |  |
|  |  |  |  |  |  |  |  |  |

**Table S4. Coefficient estimates of African wild dog iSSA models.**

|  |  |  |  |  |  |  |  |  |
| --- | --- | --- | --- | --- | --- | --- | --- | --- |
| **Model term** | **Coefficient** | **Exp. coef.** | **SE** | **RSE** | **z-score** | **p-value** | **Lower 95% CI** | **Upper 95% CI** |
|  |  |  |  |  |  |  |  |  |
| DRY SEASON |  |  |  |  |  |  |  |  |
| Grassland | 0.227 | 1.255 | 0.043 | 0.067 | 3.387 | 0.001 | 1.100 | 1.431 |
| Floodplain | -0.226 | 0.798 | 0.085 | 0.211 | -1.069 | 0.285 | 0.527 | 1.207 |
| Mixed woodland | 0.048 | 1.049 | 0.057 | 0.061 | 0.780 | 0.435 | 0.930 | 1.183 |
| Day (spline 1) | 6.189 | 487.118 | 14.164 | 2.671 | 2.317 | 0.021 | 2.594 | 91478.960 |
| Day (spline 2) | 4.111 | 61.000 | 10.828 | 3.650 | 1.126 | 0.260 | 0.048 | 77993.100 |
| Day (spline 3) | -0.281 | 0.755 | 10.643 | 3.964 | -0.071 | 0.944 | 0.000 | 1789.443 |
| Day (spline 4) | 0.102 | 1.107 | 4.797 | 1.131 | 0.090 | 0.928 | 0.121 | 10.170 |
| Step length | 0.000 | 1.000 | 0.000 | 0.000 | -5.945 | 0.000 | 1.000 | 1.000 |
| log (step length) | 0.197 | 1.218 | 0.021 | 0.033 | 5.948 | 0.000 | 1.141 | 1.299 |
| cos (turning angle) | 0.105 | 1.111 | 0.026 | 0.043 | 2.431 | 0.015 | 1.021 | 1.210 |
| Grassland * temperature (lag 0) | 0.040 | 1.040 | 0.072 | 0.059 | 0.671 | 0.502 | 0.927 | 1.168 |
| Floodplain * temperature (lag 0) | 0.167 | 1.181 | 0.200 | 0.104 | 1.597 | 0.110 | 0.963 | 1.450 |
| Mixed woodland * temperature (lag 0) | 0.017 | 1.017 | 0.114 | 0.097 | 0.172 | 0.863 | 0.841 | 1.230 |
| Grassland * temperature (lag 1) | 0.020 | 1.021 | 0.071 | 0.045 | 0.451 | 0.652 | 0.934 | 1.115 |
| Floodplain * temperature (lag 1) | -0.097 | 0.908 | 0.199 | 0.119 | -0.815 | 0.415 | 0.719 | 1.146 |
| Mixed woodland * temperature (lag 1) | -0.062 | 0.940 | 0.112 | 0.074 | -0.833 | 0.405 | 0.813 | 1.087 |
|  |  |  |  |  |  |  |  |  |
|  |  |  |  |  |  |  |  |  |
| WET SEASON |  |  |  |  |  |  |  |  |
| Grassland | 0.042 | 1.042 | 0.029 | 0.044 | 0.939 | 0.348 | 0.956 | 1.137 |
| Floodplain | -0.822 | 0.440 | 0.079 | 0.227 | -3.623 | 0.000 | 0.282 | 0.686 |
| Mixed woodland | 0.020 | 1.020 | 0.035 | 0.040 | 0.500 | 0.617 | 0.943 | 1.104 |
| Day (spline 1) | 2.313 | 10.106 | 10.067 | 3.043 | 0.760 | 0.447 | 0.026 | 3934.064 |
| Day (spline 2) | 6.658 | 778.889 | 12.675 | 3.296 | 2.020 | 0.043 | 1.219 | 497789.400 |
| Day (spline 3) | -8.457 | 0.000 | 11.533 | 3.585 | -2.359 | 0.018 | 0.000 | 0.239 |
| Day (spline 4) | 1.720 | 5.584 | 4.191 | 0.909 | 1.892 | 0.058 | 0.940 | 33.170 |
| Step length | 0.000 | 1.000 | 0.000 | 0.000 | -2.619 | 0.009 | 1.000 | 1.000 |
| log (step length) | 0.118 | 1.125 | 0.014 | 0.019 | 6.081 | 0.000 | 1.083 | 1.168 |
| cos (turning angle) | -0.011 | 0.989 | 0.017 | 0.007 | -1.545 | 0.122 | 0.974 | 1.003 |
| Grassland * temperature (lag 0) | 0.021 | 1.021 | 0.038 | 0.024 | 0.899 | 0.369 | 0.975 | 1.070 |
| Floodplain * temperature (lag 0) | 0.303 | 1.354 | 0.138 | 0.109 | 2.766 | 0.006 | 1.092 | 1.677 |
| Mixed woodland * temperature (lag 0) | -0.004 | 0.996 | 0.047 | 0.053 | -0.072 | 0.943 | 0.898 | 1.105 |
| Grassland * temperature (lag 1) | 0.022 | 1.022 | 0.038 | 0.036 | 0.593 | 0.553 | 0.951 | 1.097 |
| Floodplain * temperature (lag 1) | 0.013 | 1.013 | 0.140 | 0.155 | 0.086 | 0.931 | 0.748 | 1.373 |
| Mixed woodland * temperature (lag 1) | 0.078 | 1.081 | 0.049 | 0.048 | 1.605 | 0.109 | 0.983 | 1.188 |
|  |  |  |  |  |  |  |  |  |
|  |  |  |  |  |  |  |  |  |
|  |  |  |  |  |  |  |  |  |
| Exp. Coef = exponentiated coefficient; SE = standard error; RSE = robust standard error; CI = confidence interval | | | | | | |  |  |
|  |  |  |  |  |  |  |  |  |

**Table S5. Coefficient estimates of lion iSSA models.**

|  |  |  |  |  |  |  |  |  |
| --- | --- | --- | --- | --- | --- | --- | --- | --- |
| **Model term** | **Coefficient** | **Exp. coef.** | **SE** | **RSE** | **z-score** | **p-value** | **Lower 95% CI** | **Upper 95% CI** |
|  |  |  |  |  |  |  |  |  |
| DRY SEASON |  |  |  |  |  |  |  |  |
| Grassland | 0.197 | 1.218 | 0.038 | 0.058 | 3.412 | 0.001 | 1.087 | 1.363 |
| Floodplain | 0.203 | 1.225 | 0.044 | 0.069 | 2.942 | 0.003 | 1.070 | 1.402 |
| Mixed woodland | 0.090 | 1.094 | 0.040 | 0.061 | 1.468 | 0.142 | 0.970 | 1.235 |
| Day (spline 1) | -3.153 | 0.043 | 8.473 | 2.169 | -1.453 | 0.146 | 0.001 | 3.001 |
| Day (spline 2) | -2.462 | 0.085 | 5.829 | 1.927 | -1.278 | 0.201 | 0.002 | 3.725 |
| Day (spline 3) | -3.946 | 0.019 | 7.345 | 1.520 | -2.597 | 0.009 | 0.001 | 0.380 |
| Day (spline 4) | -2.230 | 0.108 | 2.866 | 0.737 | -3.026 | 0.002 | 0.025 | 0.456 |
| Step length | -3.28E-05 | 1.000 | 1.86E-05 | 1.76E-05 | -1.859 | 0.063 | 1.000 | 1.000 |
| log (step length) | 0.110 | 1.117 | 0.012 | 0.013 | 8.192 | < 0.001 | 1.088 | 1.147 |
| cos (turning angle) | 0.007 | 1.007 | 0.015 | 0.006 | 1.045 | 0.296 | 0.994 | 1.019 |
| Grassland * temperature (lag 0) | -0.025 | 0.976 | 0.028 | 0.014 | -1.807 | 0.071 | 0.950 | 1.002 |
| Floodplain * temperature (lag 0) | 0.050 | 1.051 | 0.049 | 0.047 | 1.064 | 0.287 | 0.959 | 1.153 |
| Mixed woodland * temperature (lag 0) | 0.052 | 1.053 | 0.040 | 0.039 | 1.346 | 0.178 | 0.977 | 1.136 |
| Grassland * temperature (lag 1) | 0.029 | 1.030 | 0.028 | 0.016 | 1.792 | 0.073 | 0.997 | 1.063 |
| Floodplain * temperature (lag 1) | -0.005 | 0.995 | 0.049 | 0.028 | -0.172 | 0.864 | 0.942 | 1.051 |
| Mixed woodland * temperature (lag 1) | -0.051 | 0.950 | 0.040 | 0.032 | -1.585 | 0.113 | 0.891 | 1.012 |
|  |  |  |  |  |  |  |  |  |
|  |  |  |  |  |  |  |  |  |
| WET SEASON |  |  |  |  |  |  |  |  |
| Grassland | 0.272 | 1.313 | 0.035 | 0.065 | 4.175 | < 0.001 | 1.155 | 1.492 |
| Floodplain | 0.236 | 1.267 | 0.050 | 0.081 | 2.913 | 0.004 | 1.080 | 1.485 |
| Mixed woodland | 0.025 | 1.025 | 0.038 | 0.050 | 0.493 | 0.622 | 0.929 | 1.132 |
| Day (spline 1) | 6.809 | 906.234 | 8.806 | 3.321 | 2.051 | 0.040 | 1.351 | 6.08E + 05 |
| Day (spline 2) | -6.596 | 0.001 | 10.654 | 2.761 | -2.389 | 0.017 | 0.000 | 0.306 |
| Day (spline 3) | -1.817 | 0.163 | 9.094 | 1.760 | -1.032 | 0.302 | 0.005 | 5.116 |
| Day (spline 4) | 1.286 | 3.617 | 3.159 | 0.531 | 2.422 | 0.015 | 1.278 | 10.234 |
| Step length | -2.29E-05 | 1.000 | 1.78E-05 | 1.08E-05 | -2.134 | 0.033 | 1.000 | 1.000 |
| log (step length) | 0.126 | 1.134 | 0.012 | 0.011 | 11.869 | < 0.001 | 1.111 | 1.158 |
| cos (turning angle) | 0.002 | 1.002 | 0.016 | 0.006 | 0.309 | 0.757 | 0.991 | 1.013 |
| Grassland * temperature (lag 0) | -0.011 | 0.989 | 0.027 | 0.017 | -0.671 | 0.502 | 0.957 | 1.022 |
| Floodplain * temperature (lag 0) | 0.011 | 1.011 | 0.052 | 0.025 | 0.428 | 0.669 | 0.962 | 1.063 |
| Mixed woodland * temperature (lag 0) | 0.010 | 1.010 | 0.039 | 0.022 | 0.464 | 0.643 | 0.968 | 1.054 |
| Grassland * temperature (lag 1) | 0.003 | 1.003 | 0.028 | 0.021 | 0.153 | 0.879 | 0.963 | 1.045 |
| Floodplain * temperature (lag 1) | 0.018 | 1.018 | 0.054 | 0.032 | 0.547 | 0.585 | 0.955 | 1.085 |
| Mixed woodland * temperature (lag 1) | -0.057 | 0.945 | 0.039 | 0.038 | -1.493 | 0.135 | 0.876 | 1.018 |
|  |  |  |  |  |  |  |  |  |
|  |  |  |  |  |  |  |  |  |
|  |  |  |  |  |  |  |  |  |
| Exp. Coef = exponentiated coefficient; SE = standard error; RSE = robust standard error; CI = confidence interval | | | | | | |  |  |
|  |  |  |  |  |  |  |  |  |

**Table S6. Summary of generalised additive mixed model results for analyses investigating changes in activity timings for lion, cheetah, and African wild dogs.**

|  |  |  |  |  |  |  |  |
| --- | --- | --- | --- | --- | --- | --- | --- |
| **Term** | **Estimate** | **EDF** | **SE** | **Ref. df** | ***t*-value** | ***F*-value** | ***P*-value** |
| CHEETAH (DRY SEASON) |  |  |  |  |  |  |  |
| (Intercept) | 0.050 |  | 0.178 |  | 0.279 |  | 0.780 |
| te(sunTime,lag0_temperature_max_scale) |  | 12.926 |  | 12.926 |  | 54.278 | < 0.001 |
| te(sunTime,lag1_temperature_max_scale) |  | 2.135 |  | 16.000 |  | 0.477 | 0.004 |
| s(day) |  | 1.000 |  | 1.000 |  | 0.737 | 0.391 |
|  |  |  |  |  |  |  |  |
| CHEETAH (WET SEASON) |  |  |  |  |  |  |  |
| (Intercept) | -0.011 |  | 0.123 |  | -0.091 |  | 0.927 |
| te(sunTime,lag0_temperature_max_scale) |  | 7.848 |  | 7.848 |  | 85.803 | < 0.001 |
| te(sunTime,lag1_temperature_max_scale) |  | 0.000 |  | 16.000 |  | 0.000 | 0.412 |
| s(day) |  | 1.000 |  | 1.000 |  | 38.848 | 0.000 |
|  |  |  |  |  |  |  |  |
| AFRICAN WILD DOG (DRY SEASON) |  |  |  |  |  |  |  |
| (Intercept) | 0.741 |  | 0.055 |  | 13.401 |  | < 0.001 |
| te(sunTime,lag0_temperature_max_scale) |  | 11.790 |  | 11.790 |  | 58.200 | < 0.001 |
| te(sunTime,lag1_temperature_max_scale) |  | 3.526 |  | 16.000 |  | 0.394 | 0.027 |
| s(day) |  | 4.561 |  | 4.561 |  | 6.214 | 0.000 |
|  |  |  |  |  |  |  |  |
| AFRICAN WILD DOG (WET SEASON) |  |  |  |  |  |  |  |
| (Intercept) | 0.952 |  | 0.058 |  | 16.300 |  | < 0.001 |
| te(sunTime,lag0_temperature_max_scale) |  | 14.577 |  | 14.577 |  | 85.521 | < 0.001 |
| te(sunTime,lag1_temperature_max_scale) |  | 0.952 |  | 16.000 |  | 0.113 | 0.123 |
| s(day) |  | 4.587 |  | 4.587 |  | 12.605 | 0.000 |
|  |  |  |  |  |  |  |  |
| LION (DRY SEASON) |  |  |  |  |  |  |  |
| (Intercept) | 1.594 |  | 0.158 |  | 10.115 |  | < 0.001 |
| te(sunTime,lag0_temperature_max_scale) |  | 14.716 |  | 14.716 |  | 20.524 | < 0.001 |
| te(sunTime,lag1_temperature_max_scale) |  | 3.653 |  | 16.000 |  | 0.380 | 0.055 |
| s(day) |  | 5.267 |  | 5.267 |  | 5.432 | < 0.001 |
|  |  |  |  |  |  |  |  |
| LION (WET SEASON) |  |  |  |  |  |  |  |
| (Intercept) | 1.455 |  | 0.211 |  | 6.891 |  | < 0.001 |
| te(sunTime,lag0_temperature_max_scale) |  | 10.065 |  | 10.065 |  | 5.217 | < 0.001 |
| te(sunTime,lag1_temperature_max_scale) |  | 7.682 |  | 16.000 |  | 1.246 | 0.001 |
| s(day) |  | 5.568 |  | 5.568 |  | 5.496 | < 0.001 |
|  |  |  |  |  |  |  |  |
| EDF = estimated degrees of freedom, SE = standard error, Ref. df = reference degrees of freedom | | | | | | | |

**Table S7. Summary of changes in activity overlap between species pairs across low (20th percentile) and warm (80th percentile) temperatures across the study period.** Mean and confidence interval values represent bootstrapped coefficients of overlap. Underlined rows indicate differences in coefficients of overlap with no overlapping confidence intervals.

| **Coefficient of overlap (mean)** | **Upper CI** | **Lower CI** | **Species dyad** | **Temperature lag** | **Temperature category** | **Season** |
| --- | --- | --- | --- | --- | --- | --- |
| *0.748* | *0.723* | *0.773* | *cheetah–lion* | *0* | *low* | *dry* |
| *0.804* | *0.774* | *0.833* | *cheetah–lion* | *0* | *high* | *dry* |
| 0.771 | 0.743 | 0.798 | cheetah–lion | 1 | low | dry |
| 0.780 | 0.750 | 0.810 | cheetah–lion | 1 | high | dry |
| *0.717* | *0.686* | *0.747* | *cheetah–lion* | *0* | *low* | *wet* |
| *0.807* | *0.777* | *0.837* | *cheetah–lion* | *0* | *high* | *wet* |
| 0.786 | 0.757 | 0.814 | cheetah–lion | 1 | low | wet |
| 0.789 | 0.758 | 0.820 | cheetah–lion | 1 | high | wet |
| 0.831 | 0.806 | 0.856 | dog–lion | 0 | low | dry |
| 0.841 | 0.817 | 0.865 | dog–lion | 0 | high | dry |
| 0.831 | 0.804 | 0.858 | dog–lion | 1 | low | dry |
| 0.856 | 0.827 | 0.886 | dog–lion | 1 | high | dry |
| 0.840 | 0.812 | 0.868 | dog–lion | 0 | low | wet |
| 0.822 | 0.794 | 0.850 | dog–lion | 0 | high | wet |
| 0.832 | 0.806 | 0.858 | dog–lion | 1 | low | wet |
| 0.822 | 0.792 | 0.851 | dog–lion | 1 | high | wet |
|  |  |  |  |  |  |  |
| CI = confidence interval | | | | | |  |


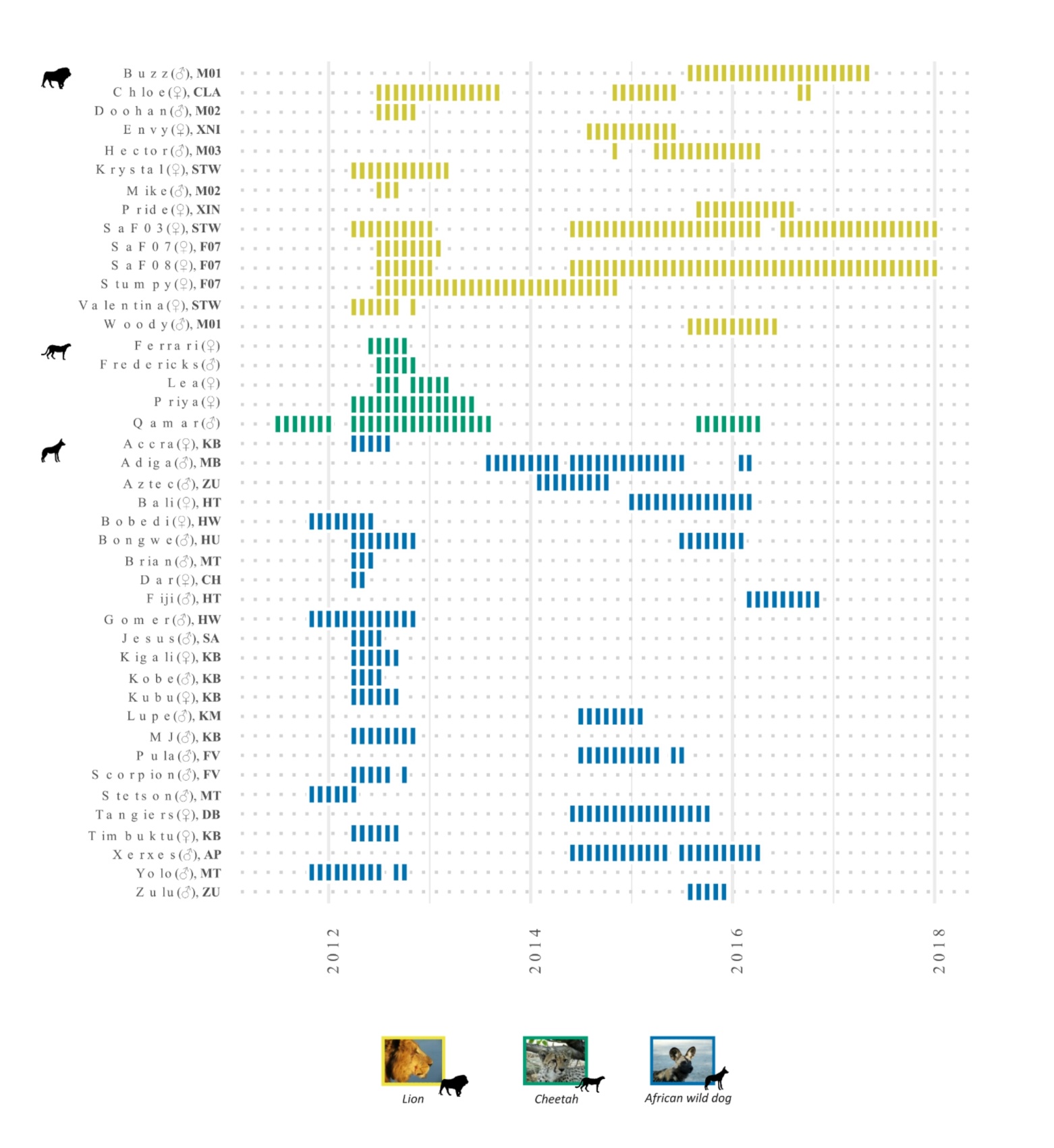


***Figure S1. Summary of collar deployment durations across species.*** The GANT chart denotes months with collar data across all collared individuals. Individual sexes are included within brackets immediately after each individual's name, followed by the social groups in bold immediately after. No social groups are included for cheetahs, as all collared individuals were solitary or part of different coalitions.


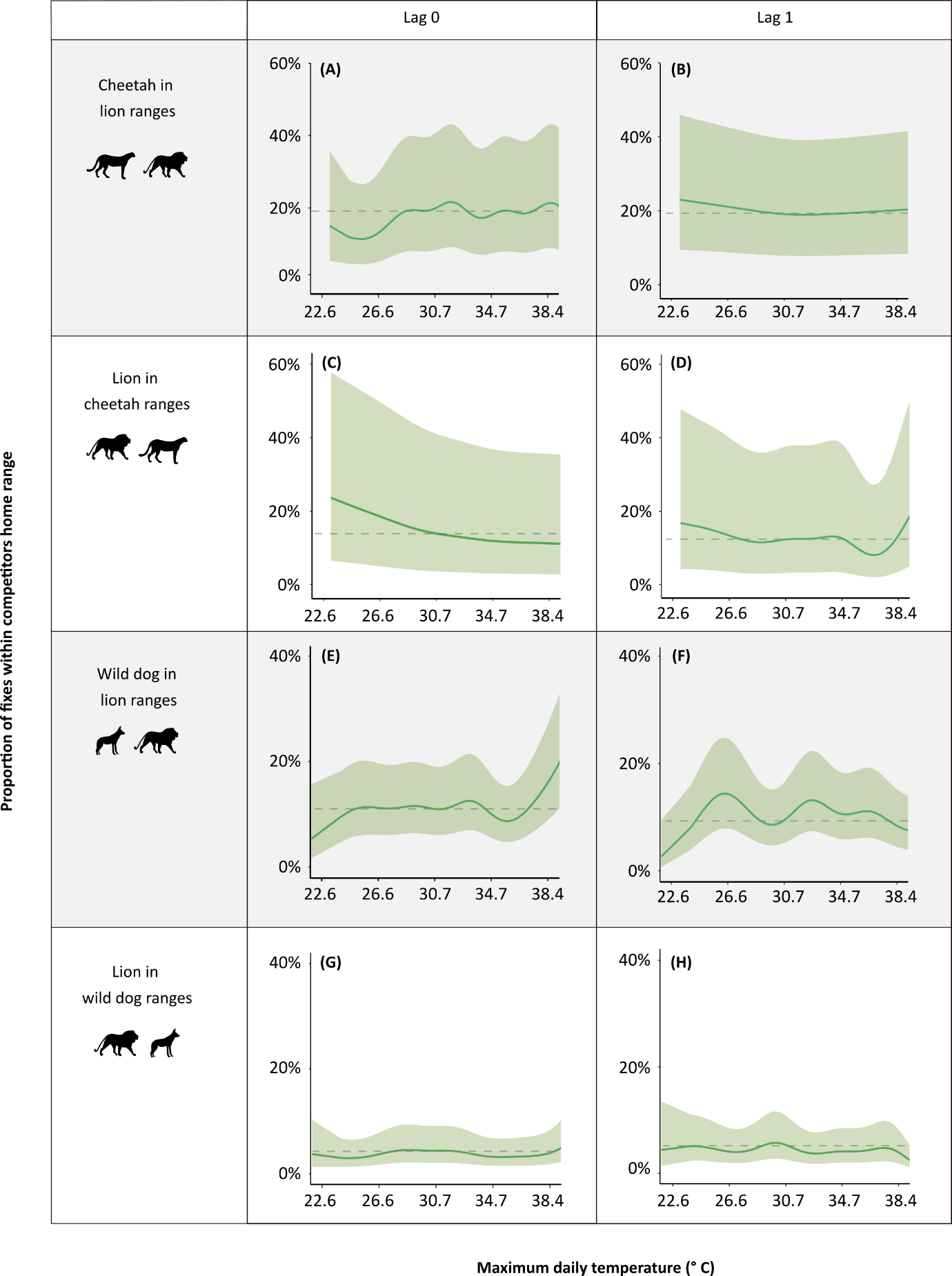


***Figure S2. Wet season proportion of GPS locations within competitor home ranges in relation to maximum daily temperatures on the day of (lag_0_) and the day prior (lag_1_).*** *Horizontal dashed lines indicate the proportion of locations within competitor home ranges at median temperature values. Maximum dail****y*** *temperature tick marks correspond approximately to -2, -1, 0, + 1, and + 2 standard deviations from the mean. Shaded ribbons represent 95% confidence intervals.*

*
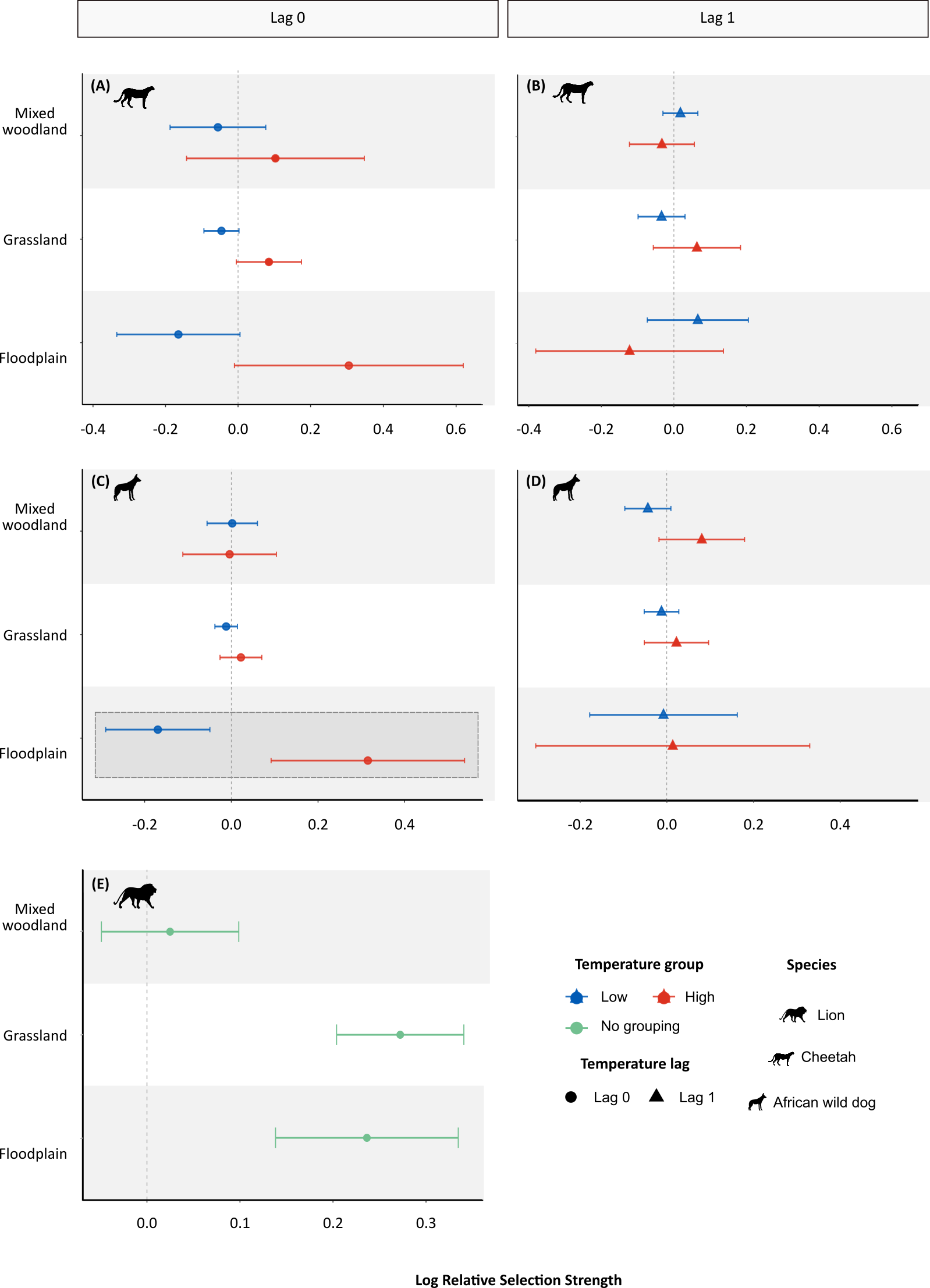
*

***Figure S3. Wet season relative selection strength estimates for mixed woodlands, grasslands, and floodplains, relative to mopane woodlands, across cool (20th percentile) and warm (80th percentile) maximum daily temperatures and across time lags for each predator species.*** *Dashed vertical lines represent no change in selection. Positive values indicate selection for that habitat and negative values represent avoidance. Bars represent 95% confidence intervals. Dashed boxes indicate habitats for which there was a statistically significant change in selection as a function of temperature.*

*
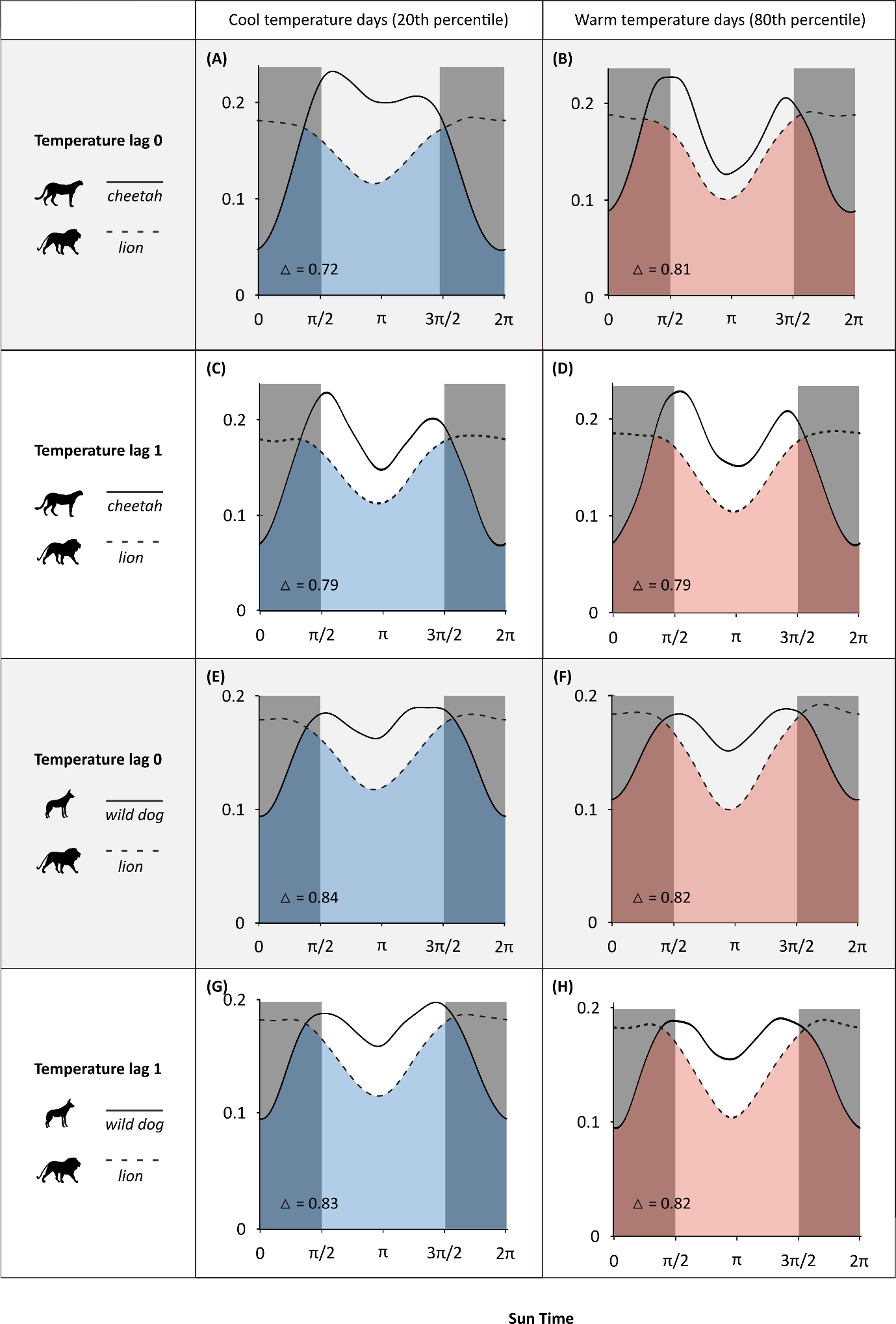
*

***Figure S4. Wet season changes in temporal partitioning between cheetahs and lions and between African wild dogs and lions across maximum daily temperatures and temperature lags.*** *Curves denote species kernel densities for activity.* *Shaded and unshaded panels on each plot delineate night and day, respectively****.*** *The x-axis denotes sun time, a transformation of clock time that anchors sunrise and sunset to π/2 and 3π/2, respectively, to control for seasonal differences in sunrise and sunset times. △ denotes the coefficient of activity overlap (where 0 indicates no overlap and 1 indicates full overlap).*
